# Progress in the Treatment of Central Nervous System Diseases Based on Nanosized Traditional Chinese Medicine

**DOI:** 10.1002/advs.202308677

**Published:** 2024-02-28

**Authors:** Jing Li, Qingyin Long, Huang Ding, Yang Wang, Dan Luo, Zhou Li, Wei Zhang

**Affiliations:** ^1^ Key Laboratory of Hunan Province for Integrated Traditional Chinese and Western Medicine on Prevention and Treatment of Cardio‐Cerebral Diseases, School of Integrated Chinese and Western Medicine Hunan University of Chinese Medicine Changsha Hunan 410208 China; ^2^ Beijing Institute of Nanoenergy and Nanosystems Chinese Academy of Sciences Beijing 101400 China; ^3^ Institute of Integrative Medicine Department of Integrated Traditional Chinese and Western Medicine Xiangya Hospital Central South University Changsha Changsha 410008 China

**Keywords:** blood‐brain barrier, central nervous system diseases, drug delivery strategies, nanocarriers, traditional Chinese medicine

## Abstract

Traditional Chinese Medicine (TCM) is widely used in clinical practice to treat diseases related to central nervous system (CNS) damage. However, the blood‐brain barrier (BBB) constitutes a significant impediment to the effective delivery of TCM, thus substantially diminishing its efficacy. Advances in nanotechnology and its applications in TCM (also known as nano‐TCM) can deliver active ingredients or components of TCM across the BBB to the targeted brain region. This review provides an overview of the physiological and pathological mechanisms of the BBB and systematically classifies the common TCM used to treat CNS diseases and types of nanocarriers that effectively deliver TCM to the brain. Additionally, drug delivery strategies for nano‐TCMs that utilize in vivo physiological properties or in vitro devices to bypass or cross the BBB are discussed. This review further focuses on the application of nano‐TCMs in the treatment of various CNS diseases. Finally, this article anticipates a design strategy for nano‐TCMs with higher delivery efficiency and probes their application potential in treating a wider range of CNS diseases.

## Graphical abstract

Traditional Chinese medicine (TCM) is widely used for central nervous system (CNS) diseases, but the blood‐brain barrier (BBB) limits its efficacy. Nanotechnology enables TCM components to cross the BBB for targeted brain treatment. This work encompasses the structure of the BBB, common TCM used to treat CNS diseases, and various nanocarriers suitable for TCM delivery. In addition, the application of nano‐TCM in the treatment of various CNS diseases has also been systematically summarized. It is expected that nano‐TCM can effectively cure a wider range of diseases in the future.

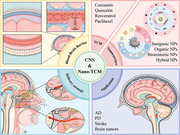

## 1 Introduction

The incidence of diseases associated with central nervous system (CNS) impairment is annually increasing. In particular, neurodegenerative diseases, such as Parkinson's disease (PD), Huntington's disease, multiple sclerosis, amyotrophic lateral sclerosis, and Alzheimer's disease (AD), as well as cerebrovascular diseases such as stroke and brain tumors, have become common in geriatric patients.^[^ ^1^ ^]^ These diseases impose an increasing economic burden on patients and society and require timely intervention and treatment. Traditional Chinese Medicine (TCM) was first used thousands of years ago to treat cerebral diseases. For example, cerebrovascular diseases are treated with the “Treatise on Cold Damage (Shang Han Lun)” and “Synopsis of the Golden Chamber (Jin Kui Yao Lue)”.^[^ ^2^ ^]^ To date, TCM and the active ingredients contained in these classical prescriptions have been used for the clinical treatment of diseases related to CNS damage, such as stroke, Parkinson's disease, or Alzheimer's disease.^[^ ^3^, ^4^, ^5^, ^6^ ^]^ Modern research has also confirmed that TCM and the effective components contained in the compound preparations can improve cerebral microcirculation disorders against damage to the nervous system and neurons.^[^ ^2^ ^]^ However, conventional water‐soluble TCM decoctions have insufficient membrane permeability and low bioavailability owing to insufficient lipid solubility and inappropriate molecular size,^[^ ^7^, ^8^ ^]^ and thus cannot effectively penetrate the blood‐brain barrier (BBB) and reach effective concentrations in the brain tissue. These issues have limited the widespread use of TCM preparations for the treatment of CNS impairment.

The BBB is a unique physiological barrier to the brain structure. As the primary protective mechanism of the CNS, the BBB forms an interface between blood in the capillaries and interstitial fluid in the brain ventricles.^[^ ^9^ ^]^ This barrier maintains and regulates CNS homeostasis by strictly controlling the movement of substances between the blood and brain, including the exchange and transport of molecules, ions, or cells. Notably, more than 98% of small‐molecule drugs and almost 100% of large‐molecule drugs cannot reach the brain through the circulatory system.^[^ ^10^ ^]^ Among them, the number of liposoluble molecules that can ultimately enter the brain is reduced by related enzymes or efflux pumps in the BBB, whereas water‐soluble molecules in the blood are blocked from entering the CNS.^[^ ^11^ ^]^ This selective transport protects the CNS from blood‐borne pathogens, toxins, inflammation, and diseases.^[^ ^12^ ^]^ The demanding “filtering” function of the BBB makes the CNS a delicate and complex tissue for the human microcirculation to penetrate, which brings significant challenges to the development of drugs delivered to the CNS.

Recently, nanotechnology has attracted considerable attention and is widely used in the diagnosis and treatment of diseases.^[^ ^13^, ^14^, ^15^, ^16^, ^17^ ^]^ Nano‐TCM is an emerging research field that combines cutting‐edge nanotechnology and TCM. This approach refers to the use of nanotechnology to prepare extracts, active ingredients, and compound formulations of TCM with particle sizes of approximately 100 nm.^[^ ^18^ ^]^ Loading or modifying TCMs and their active ingredients on nanocarriers can effectively overcome problems in the delivery of TCM, enhance solubility, improve stability and bioavailability, and prolong in vivo circulation time.^[^ ^19^ ^]^ Importantly, nano‐TCM can provide a more effective therapeutic platform for treating CNS diseases, targeting drug delivery into cells and tissues within the CNS and achieving a slow and controlled release of drugs in the brain by improving BBB penetration.^[^ ^9^, ^20^ ^]^ The application of nano‐TCM has accelerated the modernization of TCM and shows great potential for the treatment of CNS diseases. In this review, we provide a brief overview of the physiological and pathological properties of the BBB, which determine the delivery targeting strategy of nano‐TCM. We then discuss the chemical compositions, structures, and advantages of various nanocarriers with the potential to cross the BBB. In addition, the transport mechanism of nano‐TCMs across the BBB and their application in the treatment of various CNS diseases are systematically summarized. Finally, we assess the challenges and development prospects of nano‐TCMs in the clinical treatment of CNS diseases.

## 2 The Structure and Characteristics of the BBB Under Physiological and Pathological Conditions

### 2.1 Physiological Structure of BBB

**Figure 1:**
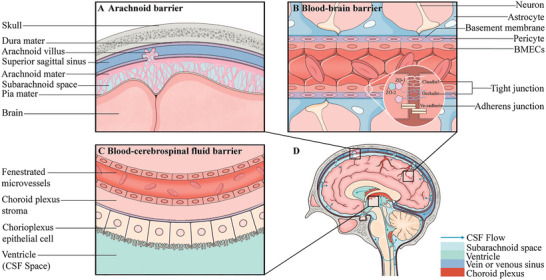
Structure of the blood‐brain barrier. The three main components of the BBB include A) the arachnoid barrier, B) the blood‐brain barrier, and C) the blood‐cerebrospinal fluid barrier, all of which, as well as the pathways of CSF flow, can be identified in D) the median sagittal section of the brain.

The BBB primarily comprises three parts: the arachnoid barrier, blood‐cerebrospinal fluid barrier (BCSFB), and BBB proper (**Figure** **1**).^[^ ^21^ ^]^ The arachnoid barrier consists of arachnoid subdural epithelial cells that segregate the extracellular fluid of the CNS (cerebrospinal fluid, CSF) from the systemic extracellular fluid (blood).^[^ ^22^ ^]^ Arachnoid villi only allow CSF to flow from the brain to the blood. Because of its relatively small total surface area and avascular nature, the arachnoid barrier seals the brain well and blocks drug delivery.^[^ ^23^ ^]^ Second, the BCSFB is composed of epithelial cells of the choroid plexus surrounding the lateral ventricle and the third and fourth ventricles of the brain.^[^ ^24^, ^25^ ^]^ CSF flows from the ventricles of the brain into the subarachnoid space, is absorbed into the superior sagittal sinus through a transvalvular pathway in the arachnoid villi, and finally returns to the systemic venous circulatory system.^[^ ^26^ ^]^ During this process, substances such as drugs or solutes enter the CNS via the choroid plexus and are swept back into the bloodstream, thereby hindering penetration into the brain. Third, the BBB is composed of the foot processes of brain capillary endothelial cells (BMECs), pericytes, astrocytes, and the complete basement membrane.^[^ ^27^ ^]^ The tight junctions formed by the arrangement of BMECs provide the barrier with high transendothelial electrical resistance,^[^ ^28^ ^]^ preventing paracellular transport and passive diffusion of most ions and molecules from the blood to the brain,^[^ ^29^, ^30^, ^31^ ^]^ thereby maintaining the integrity of the BBB. Tight junction proteins predominantly include occludin, claudin‐5, and zonula occludens proteins (ZO‐1 and ZO‐2), with claudin‐5 being the principal protein in endothelial cells.^[^ ^32^ ^]^ Additionally, adherent junctions are present between BMECs and play a crucial role in sustaining BBB integrity. Adherent junctions are composed of transmembrane glycoproteins that regulate paracellular permeability by coordinating interactions primarily through the connection of actin filaments between cells. The most representative of them is vascular endothelial cadherin (VE‐cadherin), which has been shown to be associated with cell proliferation, migration, and permeability.^[^ ^33^ ^]^ Endothelial cells rely on tight junctions and adhesive junctions to ensure the structural and functional integrity of the BBB. Pericytes are located outside BMECs and are encapsulated in the basement membrane. They participate in protein regulation through N‐cadherin and linker proteins between BMECs, maintain CNS homeostasis and BBB integrity by regulating capillary diameter to control cerebral blood flow, and regulate macrophage activity to phagocytize toxic metabolites.^[^ ^34^, ^35^ ^]^ Astrocytic endfeet are wrapped around the periphery of the pericyte layer and cover neuronal processes and blood vessels, forming a signaling network between neurons and blood vessels.^[^ ^36^ ^]^ The basement membrane is formed by the deposition of proteins secreted by these cells and is mainly involved in maintaining the stability of the microvasculature.^[^ ^37^ ^]^ The aforementioned cells integral to the regulation of BBB function are collectively termed the neurovascular unit (NVU). The NVU also includes neurons, microglia, vascular smooth muscle cells, oligodendrocytes, mast cells, and white blood cells, which regulate BBB permeability and stability.^[^ ^25^, ^38^, ^39^, ^40^ ^]^ For example, under normal conditions, leukocyte adhesion is minimized and their passage across the brain endothelium is tightly regulated. However, under pathological conditions, leukocytes can enter the BBB through certain blood vessels in the meninges and play an immunoprotective role in the CNS.^[^ ^41^, ^42^ ^]^ Notably, the BBB possesses the most extensive surface area conducive to substance exchange^[^ ^43^ ^]^ within the human body, with an average total area available for such an exchange ranging from 12 to 18 m^2^. At the same time, the size of substances that can across the BBB by passive diffusion is <25 µm.^[^ ^44^ ^]^ Moreover, there are multiple transporters, receptors, and carrier‐mediated transporters of compounds on their surfaces.^[^ ^45^, ^46^ ^]^ However, the BBB is a highly selective barrier and restricts the free entry of most substances into the CNS. TCMs and their active ingredients that do not meet the criteria for size and physicochemical properties are generally excluded. This limitation makes it challenging to achieve the expected therapeutic effects in the clinical treatment of CNS diseases. Therefore, designing drug delivery systems that can effectively cross the BBB is crucial for the successful delivery of TCM to the brain.

### 2.2 Pathological Changes of the BBB

CNS diseases, such as AD, PD, stroke, and brain tumors, are the second leading cause of death worldwide and are the main cause of disability, seriously endangering people's health and increasing economic burden.^[^ ^47^ ^]^ With the growing understanding of the physiological structure, function, and transport mechanism of the BBB, the relationship between impaired BBB function and CNS diseases has gradually become more widely studied. The physiology of the BBB undergoes various pathological changes during different CNS disease processes.

#### 2.2.1 Alzheimer's Disease

AD is the most common chronic degenerative disease of the nervous system. The number of AD patients aged ≥65 years is expected to reach 12.7 million by 2050,^[^ ^48^ ^]^ according to an Alzheimer's Association 2022 report. The clinical manifestations of AD include dementia, progressive neurocognitive dysfunction, and altered memory impairment. The patients’ independent memory, cognition, and behavior are impaired, and their quality of daily life gradually declines until death.^[^ ^49^ ^]^ Studies have shown that the major pathologic features of AD involve amyloid plaques that accumulate β‐amyloid (Aβ) peptides in blood vessels and neurofibrillary tangles of hyperphosphorylated microtubule‐bound tau.^[^ ^50^, ^51^ ^]^ The Aβ peptide is a natural metabolite comprising 36–43 amino acids formed by proteolysis of amyloid precursors.^[^ ^52^ ^]^ With the long‐term increase in Aβ peptide production and delayed clearance, Aβ aggregates to form both soluble oligomers (comprising 2–6 peptides) and intermediate neurotoxic amyloids;^[^ ^53^, ^54^, ^55^ ^]^ the aggregated fibers are arranged into β‐sheets that participate in the formation of late amyloid plaques.^[^ ^51^ ^]^ Aβ increases BBB permeability by inhibiting and disrupting the expression of tight junction proteins.^[^ ^56^, ^57^ ^]^ At the same time, the deposition of Aβ and the formation of amyloid plaques lead to cerebral amyloid angiopathy (CAA) and the degeneration of pericytes, endothelial cells, and smooth muscle cells in the brain of patients with CAA, which disrupts the integrity of the BBB.^[^ ^58^ ^]^ Tau protein is a soluble substance involved in vesicular transport and microtubule stabilization in axons. After tau is hyperphosphorylated, it dissociates from microtubules and self‐aggregates to form a paired helical filamentous structure^[^ ^59^, ^60^ ^]^ that further changes into the main component of nerve fiber tangles in pyramidal neurons.^[^ ^61^ ^]^ Its structure damages the neurons, causes synaptic dysfunction, promotes local inflammatory responses, induces cell death, and increases brain atrophy.^[^ ^62^ ^]^ Experimental studies have shown that pathological deposition of tau disrupts BBB integrity.^[^ ^63^ ^]^ In addition, peripheral cells, astrocytes, and the extracellular matrix of the basement membrane involved in the BBB are altered to various degrees in patients with AD, and these abnormal changes induce BBB dysfunction.^[^ ^64^, ^65^, ^66^, ^67^, ^68^ ^]^

#### 2.2.2 Parkinson's Disease

PD is the second most common chronic and progressive neurodegenerative disease worldwide.^[^ ^69^ ^]^ The Parkinson's Foundation reports that men are 1.5 times more likely to develop PD than women, with over 10 million individuals affected globally.^[^ ^70^ ^]^

In addition to typical motor dysfunction characterized by muscle rigidity, rest tremors, bradykinesia, and disturbances in posture and gait, such as the inability to maintain balance and shuffling gait,^[^ ^71^ ^]^ non‐motor symptoms such as dementia, hyposmia, orthostatic hypotension, constipation, memory loss, pain, depression, anxiety, urinary dysfunction, and sleep disorders may also occur.^[^ ^72^ ^]^ The progressive nature of these clinical symptoms results in increased motor disability, which significantly impairs the quality of life of patients with PD. Despite extensive research, the exact etiology and pathogenesis of the disease remain elusive.^[^ ^27^ ^]^ Pathological studies of PD suggest that their development is associated with progressive loss of dopaminergic neurons in the substantia nigra, deposition of neurotoxic oligomers composed of misfolded α‐synuclein (α‐syn), excessive reactive oxygen species (ROS) production, and neuroinflammation.^[^ ^73^, ^74^, ^75^, ^76^ ^]^ Inflammatory mediators released during pathological processes alter BBB permeability by affecting the arrangement and expression of tight junction proteins on BMECs.^[^ ^77^ ^]^ At the same time, P‐glycoprotein‐mediated active transport is downregulated, which inhibits the CNS efflux function that transports toxic substances from the intracellular space to the extracellular space.^[^ ^9^ ^]^ The dysfunction of the BBB leads to the degeneration of dopaminergic neurons in the substantia nigra and disrupts dopamine production.^[^ ^78^, ^79^, ^80^ ^]^ Dopamine, as a neurotransmitter that transmits chemicals, is involved in the regulation of various functions of the CNS, especially in coordination and control of motor function.^[^ ^81^ ^]^ Decreased dopamine secretion is closely associated with motor dysfunction in PD.

#### 2.2.3 Stroke

Stroke is the second most common cause of death globally and the primary cause of prolonged disability.^[^ ^82^, ^83^, ^84^ ^]^ The American Stroke Association reported that stroke is the leading cause of disability in the United States.^[^ ^85^ ^]^ Moreover, the Global Burden of Disease Study revealed that, in 2019, there were 12.2 million new cases of stroke and 6.55 million fatalities associated with stroke.^[^ ^84^ ^]^ Strokes are classified into hemorrhagic and ischemic types, based on their distinct etiologies. Hemorrhagic stroke is caused by cerebral blood vessel rupture and bleeding, whereas ischemic stroke is caused by vascular obstruction.^[^ ^86^ ^]^ Among these, ischemic strokes account for 80–85% of cases, where the occlusion of blood vessels compromises cerebral blood supply, leading to brain tissue damage.^[^ ^86^ ^]^ Clinical symptoms vary from localized to widespread neurological dysfunction, including dizziness, hemianopia, hemiplegia, aphasia, sensory disturbances, and consciousness disorders, depending on the extent of ischemia.^[^ ^87^ ^]^ Recent studies have identified the pathophysiological mechanisms of ischemic stroke.^[^ ^86^ ^]^ Neurons in the brain lack the ability to store energy independently and often rely on cerebrovascular transport to provide energy. When vascular occlusion causes a decrease in cerebral blood flow to downstream regions, the corresponding supply of glucose and oxygen in the cerebral blood vessels is also reduced, thereby affecting neurons and initiating an ischemic cascade of inflammation and cell death.^[^ ^88^, ^89^, ^90^ ^]^

Research has demonstrated that the integrity of the BBB is disrupted and its permeability is significantly increased during cerebral ischemia, both in humans and animal models.^[^ ^91^ ^]^ The two most relevant factors involved in the disruption of BBB are tissue plasminogen activators (tPAs) and matrix metalloproteinases (MMPs). tPAs are serine proteases that activate the conversion of plasminogen to active plasmin.^[^ ^92^ ^]^ In the ischemic brain parenchyma, vascular and parenchymal tPAs interact with various components in the NVU to cause dysfunction of tight junctions, shedding of astrocytic endfeet, and degradation of basement membranes, which not only increases the risk of hemorrhagic transformation and angiogenic edema but also destroys the integrity and permeability of the BBB.^[^ ^93^, ^94^, ^95^, ^96^, ^97^, ^98^ ^]^ MMPs are proteases that cleave components of the extracellular matrix.^[^ ^99^ ^]^ Expression of MMPs is often difficult to detect under physiological conditions, but several overexpressed MMPs, such as MMP‐2, MMP‐3, MMP‐7, and MMP‐9, can be detected in ischemic stroke.^[^ ^100^, ^101^, ^102^, ^103^, ^104^, ^105^ ^]^ MMP‐2 and MMP‐9 have been shown to affect the permeability of BBB by cleaving claudin‐5 to disable the tight junctions of the BMECs.^[^ ^106^ ^]^ Additionally, many studies have shown that MMP‐9 is involved not only in the process of ischemia but also in the destruction of the BBB.^[^ ^103^, ^107^ ^]^ MMP‐9 can cleave the tight junction protein, ZO‐1, and also participates in the degradation of basement membrane proteins.^[^ ^88^ ^]^

#### 2.2.4 Brain Tumors

Cancer is widely recognized as a leading cause of death worldwide and poses a significant threat to individual health and societal stability. Brain tumors are associated with high morbidity and mortality due to their location and aggressive growth characteristics.^[^ ^108^ ^]^ Data from the National Cancer Institute indicate that there are 4.5 deaths per 100,000 individuals annually attributed to brain and other nervous system cancers, comprising an estimated 3% of all cancer fatalities in 2022.^[^ ^109^ ^]^ The development of brain tumors is typically linked to intrinsic genetic factors and extrinsic environmental influences^[^ ^110^ ^]^ and can be divided into two main types: primary brain tumors that occur in the brain parenchyma and secondary brain tumors that metastasize to the cranium.^[^ ^111^ ^]^ Headaches (30%) and seizures (50–80%) are common symptoms in the majority of brain tumor patients.^[^ ^112^, ^113^ ^]^ Moreover, rapidly growing tumors in the brain tend to cause symptoms of increased intracranial pressure (15%) such as persistent nocturnal aggravation of headaches, morning nausea and vomiting, and papilledema, among others.^[^ ^113^ ^]^ In addition to the aforementioned systemic clinical manifestations, tumors located in the brain can cause significant regional neurological damage, affecting the corresponding anatomical locations in the body and resulting in local clinical manifestations. For example, tumors located in the corpus callosum and prefrontal or temporal lobes can cause cognitive deficits such as short‐term memory impairment and/or personality and mood changes. Subtentorial tumors can cause ataxia, balance disorders, long tract signs, or cranial nerve palsy. Tumors located in the parietal lobe may cause hemiplegia, sensory disturbances, and spatial disorientation. Tumors located in the frontal lobe can cause dysphagia. Visual disturbances can be caused by tumors located in the parietal, temporal, and occipital lobes or those that compress the optic nerve.^[^ ^114^ ^]^

Owing to the confined internal space of the brain, the progressive invasion of primary brain tumors or secondary brain metastases directly disrupts neuronal function through the growth and compression of blood vessels, leading to vascular dysfunction and structural impairment.^[^ ^115^ ^]^ However, this does not affect the nutritional supply of tumor cells, and in addition to the existing blood vessels, there are newly generated tumor vessels and other vascular supply mechanisms that meet their nutritional requirements.^[^ ^28^, ^116^, ^117^ ^]^ Although tumors compromise the integrity and permeability of the BBB, specialized endothelial cells situated between tumor cells and vessels form an alternative barrier known as the blood‐brain tumor barrier (BBTB).^[^ ^118^ ^]^ This BBTB has functions similar to those of the BBB, such as the expression of active efflux transporters in tumor cells, which prevent the entry of certain foreign substances.^[^ ^119^ ^]^ In light of the presence of the BBB and BBTB, TCM agents used for the treatment of the abovementioned CNS damage‐related diseases cannot enter the brain and reach effective concentrations.

### 2.3 Impact of BBB Changes on TCM Delivery

Under physiological conditions, the stability and integrity of the BBB are guaranteed by the NVU and intercellular connections, which create a formidable obstacle to the effective delivery of TCM. First, tight junctions between endothelial cells restrict the free diffusion of TCM. Second, even TCM molecules that enter endothelial cells may have active exclusion mechanisms, such as drug efflux mediated by P‐glycoprotein. Moreover, the physical and chemical properties of most TCM molecules may pose limitations, such as size, charge, and solubility, which may not meet BBB permeation standards. In particular, it is difficult for large TCM molecules and hydrophobic compounds to cross the BBB.

Under pathological conditions, alterations in the physiological structure and function of the BBB have varying effects on drug delivery. Disruptions in tight junctions and integrity, as mentioned in various CNS diseases in Section 2.2, lead to changes in permeability, with higher permeability in the BBTB than in the BBB.^[^ ^120^ ^]^ Nevertheless, large TCM molecules still face challenges in overcoming this barrier. Additionally, the lack of targeting specificity and low bioavailability of TCM makes it difficult, even upon entry into the brain, to accumulate and maintain effective therapeutic concentrations in diseased areas. However, pathological changes in BBB transporters have diverse effects on drug delivery. For example, brain tumor blood vessels often overexpress certain receptors, allowing active transport for targeted delivery to brain tumor tissues.^[^ ^121^ ^]^ Conversely, the absence of ANKS1A in AD reduces the levels of low‐density lipoprotein receptor‐related protein 1, complicating the strategies for active transport.^[^ ^122^ ^]^

The emergence of nano‐TCMs provides an opportunity to overcome this obstacle. The combination of nanotechnology and TCM can improve the targeting and therapeutic effects of TCM, bringing hope for the cure of CNS diseases.

## 3 TCM for CNS Diseases

Since 200 AD, TCM has appeared in reports of medical cases and ancient books for the treatment of CNS‐related disorders. Many of the prescriptions and herbal formulations mentioned in these records have persisted through the ages, playing a crucial and indispensable role in the clinical healthcare systems in China and Chinese communities. In addition to its clinical applications, TCM is also an important source of modern drug development.^[^ ^123^, ^124^ ^]^ Drawing on ancient TCM theories and combining them with advanced modern technologies, numerous active compounds extracted from traditional herbs have been shown to possess neuroprotective effects.^[^ ^5^, ^125^ ^]^ The pharmacological mechanisms of these active compounds against CNS diseases have been continuously studied and verified, and have been applied in clinical treatments.^[^ ^126^, ^127^, ^128^ ^]^ In the following sections, we have selected several representative TCM natural compounds widely used in both clinical treatments and scientific research of CNS diseases. This summary includes their sources, therapeutic effects, mechanisms of action, and existing delivery challenges.

### 3.1 Active Ingredients of TCM for CNS Diseases

#### 3.1.1 Curcumin

Curcumin (CUR) is a natural phenolic compound extracted from plants belonging to the Araceae family, such as *Acorus tatarinowii*, or those belonging to the Zingiberaceae family, including turmeric, zedoary, and Curcuma. It is a diketone with a chemical formula of C_21_H_20_O_6_ (chemical name 1,7‐bis[4‐hydroxy‐3methoxyphenyl]‐1,6‐heptadiene‐3,5‐dione).^[^ ^129^ ^]^ Curcumin possesses a wide range of pharmacological activities, including anti‐inflammatory, antioxidant, antiviral, anti‐infective, antitumor, anti‐liver fibrosis, and lipid‐lowering effects.^[^ ^129^ ^]^ It can be used to treat various CNS‐related disorders.

CUR exerts anti‐AD effects through various mechanisms. It can inhibit the generation of Aβ, reduce plaque deposition, alleviate Aβ‐induced damage to nerve cells (such as PC12 cells), regulate tau protein phosphorylation, promote clearance, and diminish the formation of neurofibrillary tangles.^[^ ^130^, ^131^, ^132^, ^133^, ^134^ ^]^ Additionally, CUR can bind with Aβ, not only inhibiting fibril formation by disrupting Aβ peptide aggregation but also serving as an early diagnostic reagent for the detection of plaque deposition in the brain due to its fluorescent properties.^[^ ^135^, ^136^ ^]^

CUR effectively alleviates PD progression. It primarily improves the motor and behavioral impairments in PD mice by reducing the deposition of phosphorylated α‐syn and the death of dopaminergic neurons in the substantia nigra.^[^ ^137^, ^138^ ^]^ In terms of antioxidation, it not only reduces the generation of mitochondrial ROS but also through the phenolic hydroxyl and alkyl CH_2_ groups in its structure, binds with ROS to neutralize and eliminate them by capturing hydrogen atoms, thereby reducing oxidative stress in the brain.^[^ ^139^, ^140^ ^]^

CUR has also significantly contributed to the prevention and treatment of ischemic stroke. It exerts anti‐inflammatory effects by promoting the polarization of M2 microglial cells and inhibiting the activation of the nuclear factor kappa‐B (NF‐κB) signaling pathway.^[^ ^141^, ^142^ ^]^ It plays an anti‐apoptotic role by regulating the secretion of apoptosis‐related proteins and blocking endoplasmic reticulum stress‐related apoptotic pathways.^[^ ^143^, ^144^, ^145^ ^]^ Additionally, it provides neuroprotection during cerebral ischemia‐reperfusion injury (CIRI) by modulating autophagy, protecting astrocytes, and maintaining the structure and function of the BBB.^[^ ^146^, ^147^, ^148^ ^]^

CUR also possesses antitumor properties. First, by inhibiting the Janus kinase (JAK)/signal transducer and activator of transcription 3 (STAT3) signaling pathway, CUR effectively impedes the migration and invasion of glioblastoma (GBM) cells and inhibits the survival and proliferation of tumor cells.^[^ ^149^, ^150^ ^]^ Second, CUR induces cell cycle arrest in the G2/M phase through various mechanisms that disrupt the growth and proliferation of glioma cells.^[^ ^151^, ^152^, ^153^ ^]^ Furthermore, CUR exerts its anticancer effects by inhibiting the mammalian target of rapamycin (mTOR) signaling pathway, activating autophagy, and simultaneously eliminating the tumorigenicity of GBM stem cells, reducing the risk of recurrence.^[^ ^154^, ^155^ ^]^ Finally, CUR induces apoptosis in GBM cells by upregulating the expression of pro‐apoptotic proteins, such as caspase‐7, caspase‐8, caspase‐3, and caspase‐9.^[^ ^156^ ^]^ These multiple mechanisms collectively demonstrate the multifaceted effects of CUR in combating tumors.

#### 3.1.2 Quercetin

Quercetin (QCT) is widely distributed and is mainly found in plants and human foods such as apples, onions, tomatoes, red‐leaf lettuce, asparagus, and herbs.^[^ ^157^ ^]^ It is a flavonoid with the chemical formula C_15_H_10_O_7_ name (3,3′,4′,5,7‐pentahydroxyflavone).^[^ ^158^ ^]^ QCT exhibits diverse pharmacological properties including anti‐allergic, anti‐inflammatory, antimicrobial, anticancer, antiviral, antioxidant, and neuroprotective activities.^[^ ^159^, ^160^ ^]^ It is believed to be beneficial for combating various CNS disorders.

QCT primarily exerts its anti‐AD effects through anti‐cholinergic, anti‐inflammatory, antioxidant, and anti‐neurotoxic protein aggregation activities. QCT improves cognitive function by inhibiting the activity of acetylcholinesterase and protects astrocytes by modulating the release of neuroinflammatory cytokines, such as IL‐1β and TNF‐α.^[^ ^161^, ^162^, ^163^ ^]^ QCT can directly neutralize highly reactive oxygen species and reduce oxidative stress levels by activating the antioxidant pathway, nuclear factor‐like 2 (Nrf‐2), or regulating the transcription of antioxidant factors, such as paraoxonase‐2.^[^ ^164^, ^165^, ^166^ ^]^ Additionally, QCT can slow down the progression of AD by inhibiting tau protein phosphorylation and the aggregation of Aβ peptides.^[^ ^166^, ^167^ ^]^

QCT also has potentially beneficial effects on PD by preventing neuronal damage and death through multiple mechanisms. Specifically, QCT can improve neural inflammation by inhibiting the secretion of inflammatory factors such as TNF‐α, IL‐1β, and IL‐6.^[^ ^168^ ^]^ Moreover, it plays a neuroprotective role by regulating the levels of antioxidant enzymes, such as glutathione peroxidase and superoxide dismutase (SOD).^[^ ^169^ ^]^ Additionally, QCT enhances both motor and cognitive functions by modulating dopamine metabolism in the striatum.^[^ ^170^, ^171^ ^]^ Furthermore, it can inhibit α‐syn fibrillation, effectively slowing disease progression.^[^ ^172^ ^]^ Through these diverse mechanisms of action, QCT provides extensive and comprehensive support for the treatment of PD.

In the treatment of ischemic stroke, QCT primarily exerts neuroprotective effects through anti‐inflammatory and antioxidant stress responses. In terms of anti‐inflammatory action, QCT can upregulate the secretion of anti‐inflammatory cytokines (such as IL‐4, IL‐10) and downregulate the release of proinflammatory cytokines (such as IL‐6, IL‐1).^[^ ^173^ ^]^ QCT also facilitates the phenotypic transition of microglial cells from proinflammatory M1 to anti‐inflammatory M2.^[^ ^174^ ^]^ Regarding antioxidant effects, QCT can alleviate reperfusion‐induced neurotoxicity by downregulating extracellular signal‐regulated kinase (ERK) and protein kinase B (PKB, also known as AKT) phosphorylation.^[^ ^175^ ^]^ QCT also downregulates thioredoxin expression during focal cerebral ischemia and glutamate‐induced neuronal cell death.^[^ ^176^ ^]^ Additionally, QCT enhances neuronal activity in the prefrontal cortex, amygdala, and hippocampus by inhibiting astrocytic and microglial reactivation.^[^ ^177^ ^]^

QCT exerts its anticancer effects through various tumor‐related mechanisms, including oxidative stress, cell proliferation, apoptosis, cell cycle, and metastasis.^[^ ^178^ ^]^ Through the AKT/ERK/Caspase‐3 signaling pathway, QCT induces autophagy and apoptosis in glioma cells.^[^ ^179^ ^]^ In GBM cells, QCT induces mitochondria‐mediated apoptosis and protective autophagy and inhibits proliferation by inducing cell cycle arrest.^[^ ^180^ ^]^ Additionally, QCT downregulates phospholipase D1, indirectly inhibiting the activation of MMP‐2, thereby impeding the proliferation, migration, and invasion of GBM cells.^[^ ^181^ ^]^ Importantly, QCT not only enhances the sensitivity of brain tumor cells to radiotherapy and chemotherapy but also prevents the efflux of transport proteins, providing a synergistic effect for the subsequent treatment of drug‐resistant brain cancer.^[^ ^182^, ^183^, ^184^ ^]^

#### 3.1.3 Resveratrol

Resveratrol (RES), first discovered in *Veratrum grandiflorum*, is produced by plants such as grapes, Japanese knotweed, cassia, blueberries, and peanuts and is an antitoxin when stimulated.^[^ ^185^, ^186^, ^187^ ^]^ Its chemical formula is C_14_H_12_O_3_ and its chemical structure is 3,5,4′‐trihydroxybenzene, belonging to the category of non‐flavonoid polyphenol compounds.^[^ ^188^ ^]^ RES has been proven to possess various pharmacological activities beneficial to health, including anti‐inflammatory, antioxidant, anticancer, cardiovascular, and neuroprotective effects.^[^ ^189^, ^190^ ^]^

RES exerts neuroprotective effects against the pathological processes of AD through various mechanisms.^[^ ^191^ ^]^ For instance, as a SIRT1 activator, RES can mitigate the impact of microglia‐dependent Aβ toxicity on neurons by inhibiting the NF‐κB signaling pathway.^[^ ^192^ ^]^ Additionally, RES effectively reduces the generation of ROS through various antioxidant mechanisms and repairs mitochondrial dysfunction, thereby lowering Aβ‐induced lipid peroxidation and neurotoxicity levels.^[^ ^193^, ^194^, ^195^ ^]^ Furthermore, RES plays a crucial role in alleviating the cytotoxic effects of nitric oxide (NO) and inhibiting inflammatory cascades, thereby producing neuroprotective effects.^[^ ^196^ ^]^ RES also prevents nerve cells from taking up extracellular tau oligomers, thereby improving cognitive impairments in mice by halting tau phosphorylation, preventing synaptic loss, and inhibiting the progression of neuroinflammation.^[^ ^197^ ^]^

The impact of RES in the prevention and treatment of PD is profound.^[^ ^198^, ^199^ ^]^ Research suggests that RES plays a crucial role in neuroprotection by activating the Nuclear factor erythroid2‐related factor 2 (Nrf2) signaling pathway and counteracting oxidative stress. Its antioxidant mechanisms include not only direct scavenging of ROS but also augmentation of endogenous antioxidant levels.^[^ ^200^ ^]^ Moreover, RES can decelerate neuroblast apoptosis and mitigate motor dysfunction induced by 6‐hydroxydopamine in PD rats through activation of the phosphoinositide 3‐kinase (PI3K)/AKT signaling pathway.^[^ ^201^ ^]^ In addition to these mechanisms, RES demonstrates anti‐inflammatory and antioxidant properties, contributing to the amelioration of motor and cognitive dysfunctions in mice with PD. This is achieved through the reduction of α‐syn and oligomer levels, inhibition of α‐syn aggregation, and alleviation of cellular toxicity.^[^ ^202^ ^]^

RES also exhibits protective activity against ischemic stroke.^[^ ^188^, ^203^ ^]^ For instance, RES can reduce inflammation by downregulating the Toll‐like receptor 4 (TLR4) signaling pathway, thereby decreasing brain damage and repairing the damaged BBB in focal cerebral ischemia in rats.^[^ ^204^ ^]^ RES enhances the survival of hypoxic neurons in stroke rats by restoring mitochondrial metabolism, promoting neuronal differentiation, and improving neurological function.^[^ ^205^ ^]^ Furthermore, in neonatal rats with hypoxic‐ischemic brain injury, RES effectively mitigated inflammation and oxidative stress levels by upregulating the Nrf2/Heme Oxygenase‐1 (HO‐1) signaling pathway, demonstrating its neuroprotective effects.^[^ ^206^ ^]^

The anticancer properties of RES have been reported in numerous studies.^[^ ^207^, ^208^, ^209^ ^]^ Research indicates that RES can induce intrinsic apoptosis and autophagy in neuroblastoma cells by activating the endoplasmic reticulum stress‐induced intracellular reactive oxygen species (iROS) axis. It effectively inhibits Rho‐dependent cell migration, leading to tumor cell death and extending the survival of neuroblastoma mice.^[^ ^210^ ^]^ In GBM, RES effectively inhibits the proliferation and invasion of tumor cells by modulating AKT and p53.^[^ ^211^ ^]^ Additionally, RES serves as a sensitizer to address the challenging issue of chemotherapy resistance.^[^ ^212^, ^213^ ^]^ For instance, RES enhances the sensitivity of GBM to temozolomide by downregulating STAT3 and O6‐methylguanine‐DNA methyltransferase (MGMT) levels associated with alkylating agent resistance, effectively attenuating tumor growth.^[^ ^214^ ^]^ RES can also increase the chemosensitivity of GBM cells to doxorubicin by inhibiting the PI3K/AKT and P‐glycoprotein pathways.^[^ ^215^ ^]^

#### 3.1.4 Paclitaxel

Paclitaxel (PTXL), chemical formula C_47_H_51_NO_14_, was initially isolated and purified from the bark of *Taxus brevifolia*.^[^ ^216^ ^]^ The chemical structure of PTXL is complex and features a highly oxygenated tetracyclic framework bridging a bicyclo[5.3.1]undecane ring system. The key structural elements responsible for its primary anticancer activity include the A‐ring, C2 benzoyl group, C13 side chain, and oxygen‐substituted ring.^[^ ^217^, ^218^ ^]^ It belongs to the diterpenoid class of compounds.^[^ ^219^ ^]^

PTXL, as one of the most successful natural anticancer drugs in the last 50 years, primarily exerts its anticancer mechanism by stabilizing microtubule polymers through binding to β‐tubulin. This interference disrupts the mitotic process in the G2/M phase of cells, preventing the division and proliferation of tumor cells and ultimately leading to cancer cell death.^[^ ^220^ ^]^ Additionally, PTXL demonstrates significant anticancer effects through various mechanisms, such as inducing autophagy, promoting cell apoptosis, and inhibiting tumor angiogenesis.^[^ ^218^, ^219^ ^]^

As a first‐line anticancer drug, PTXL is commonly used in combination with other anticancer medications to treat brain cancer. For instance, the conjugation of linoleic acid with PTXL demonstrates low cytotoxicity, high cellular absorption rates, and improved efficacy against gliomas.^[^ ^221^ ^]^ Polymeric nanomedicines loaded with doxorubicin and PTXL significantly inhibited GBM growth and effectively ameliorated motor function impairment in rats.^[^ ^222^ ^]^ The co‐administration of PTXL and temozolomide has been demonstrated to elicit a more pronounced antitumor effect against GBMs by modulating the Wnt/β‐Catenin signaling pathway.^[^ ^223^ ^]^

### 3.2 Limitations of TCM for CNS Diseases

TCM has gained widespread recognition for its therapeutic effectiveness in the treatment of CNS diseases that has persisted since ancient times to the present day. With the continuous development of science and technology, in addition to the representative drugs mentioned above, research has confirmed the remarkable achievements of TCM in regulating neural function, repairing nerve damage, and improving quality of life. In the treatment of CNS diseases, TCM not only adheres to the traditional wisdom accumulated over centuries but also gradually reveals its unique contributions in the field of modern medical research.^[^ ^5^, ^6^ ^]^ However, traditional formulations, primarily in the form of decoctions, are inconvenient both in preparation and administration and exhibit notable instability. On the research front, the complex and diverse composition of traditional Chinese herbal compounds, along with their unclear mechanisms of action, poses challenges. Consequently, the contemporary research focus in TCM has shifted toward exploring natural active ingredients extracted from various plants or minerals. Common Chinese herbal compounds include flavonoids, polyphenols, polysaccharides, glycosides, saponins, alkaloids, terpenes, and sesquiterpenes.^[^ ^224^ ^]^ Within the natural pharmaceutical treasure trove of TCM, numerous natural compounds, such as baicalin, arsenic trioxide, berberine, muscone, puerarin, and ginkgolides, are also found to be applicable in the treatment of CNS diseases.^[^ ^225^, ^226^, ^227^, ^228^, ^229^, ^230^, ^231^, ^232^, ^233^ ^]^ However, many of these natural compounds exhibit unfavorable physicochemical properties, including low solubility, poor water‐solubility, low oral bioavailability, poor stability, rapid hepatic metabolism, low intestinal absorption, fast systemic clearance, short half‐life, and potential hepatorenal toxicity.^[^ ^7^, ^8^, ^234^, ^235^, ^236^ ^]^ Moreover, their large molecular size hampers their ability to penetrate the BBB, limiting their accumulation within brain tissues to achieve and maintain effective therapeutic concentrations. These limitations significantly constrain the clinical application of TCM for the treatment of CNS diseases. Modern advanced technologies, such as nanotechnology, have become indispensable to maximize the potential value of TCM. The introduction of nanotechnology addresses various challenges and offers several advantages, including: 1) altering the traditional administration routes of TCM and diversifying TCM formulations; 2) achieving controlled and targeted drug delivery to reduce the toxic side effects of TCM; 3) enhancing the existing therapeutic effects of TCM and introducing new pharmacological activities; 4) increasing the solubility and stability of TCM, improving bioavailability, reducing the required dosage, and conserving TCM resources; and 5) improving its pharmacokinetics and enhancing BBB permeability.^[^ ^19^, ^234^, ^237^, ^238^, ^239^ ^]^ In conclusion, the integration of modern advanced technologies, particularly nanotechnology, is pivotal for unlocking the full potential of TCM. This not only addresses existing challenges, but also brings forth a myriad of advantages, revolutionizing the administration, delivery, and overall efficacy of TCM, thus contributing to its optimal utilization and resource conservation.

## 4 Nanocarriers of Nano‐TCM

The preparation of nano‐TCM is generally categorized into two types: one is to use nano‐processing technology to nanosize the active ingredients of TCM, and the other, the most commonly used strategy, is to combine the active ingredients of TCM with nanocarriers. In the first approach, the drug is nanosized, and the specific surface area of the herb particles is increased after ultrafine pulverization. This process generates many surface‐active atoms that can alter the physicochemical properties and biological activity of the drug, thereby improving its dissolution rate and overall efficacy. However, it is worth noting that the use of nanocarriers provides superior bioavailability and targeting compared to drug nanosizing alone. Therefore, this review focuses on the more promising route for combining the active ingredients of TCM with nanocarriers. Nanocarriers are drug delivery systems on a nanoscale (1–100 nm) that are commonly used to modulate the pharmacokinetic and pharmacodynamic characteristics of drugs.^[^ ^240^ ^]^ Additionally, the unique physical properties of nanomaterials can enhance drug delivery and synergize drug treatments.^[^ ^241^ ^]^ Nanocarriers carrying various functional drug molecules have been widely used for the diagnosis and treatment of CNS diseases at the molecular, cellular, and individual levels. Nanocarriers can be divided into three categories based on their chemical composition: inorganic nanocarriers, organic nanocarriers, and inorganic/organic hybrid nanocarriers.

### 4.1 Inorganic Nanocarriers

**Figure 2:**
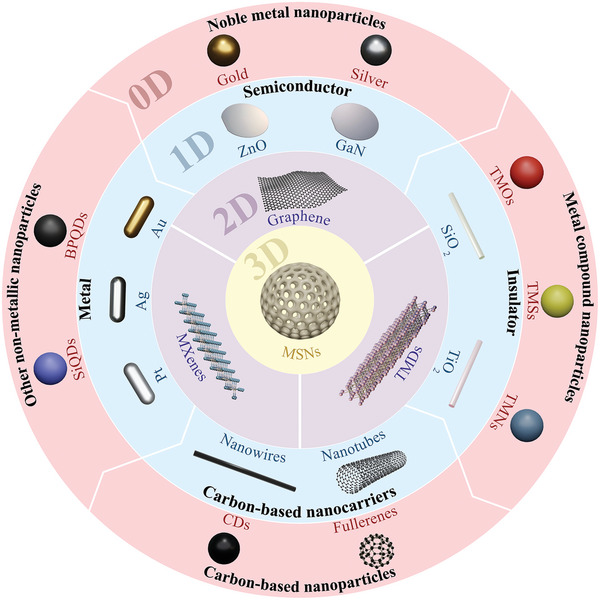
Schematic diagram of the classification of inorganic nanocarriers, including 0D, 1D, 2D, and 3D nanocarriers. Notes:TMOs: Transition metal oxides; TMSs: Transition metal sulfides; TMNs: Transition metal nitrides; SiQDs: Silicon quantum dots; BPQDs: Black phosphorus quantum dots.

In recent years, inorganic nanocarriers have attracted considerable attention owing to their high stability, easy preparation, and unique physical and chemical properties (magnetic, electrical, optical, and thermal).^[^ ^242^ ^]^ Inorganic nanocarriers play important roles in marker detection, bioimaging, targeted delivery, and disease treatment.^[^ ^243^, ^244^, ^245^ ^]^ According to the topological structure, inorganic nanocarriers can be divided into 0D, 1D, 2D, and 3D subtypes (**Figure** **2**).^[^ ^246^ ^]^ Common 0D nanocarriers include noble metal nanoparticles (e.g., gold and silver), metal compound nanoparticles (e.g., transition metal oxides/sulfides/nitrides), carbon‐based nanoparticles (e.g., carbon dots, fullerenes), and other nonmetallic nanoparticles (e.g., silicon quantum dots, black phosphorus quantum dots).^[^ ^247^, ^248^ ^]^ Typical representatives of 1D nanocarriers are metal‐(such as Au, Ag, Pt)/semiconductor‐ (such as ZnO, GaN)/insulator‐(such as SiO₂, TiO₂)/carbon‐based nanowires and nanotubes.^[^ ^249^ ^]^ Emerging 2D nanocarriers include graphene and its derivatives, transition metal dichalcogenides (TMDs), and transition metal carbon‐nitrides (e.g., MXenes).^[^ ^250^, ^251^ ^]^ A commonly used 3D nanocarrier in biomedicine is mesoporous silicon nanoparticles (MSNs).^[^ ^252^ ^]^ It is worth noting that there are currently relatively few studies on nano‐TCM based on inorganic nanocarriers for the treatment of CNS diseases, mainly focusing on the following materials:

#### 4.1.1 Noble Metal Nanoparticles

Noble metal nanoparticles, primarily Au and Ag nanoparticles, are the most common drug carriers (Figure 2).^[^ ^253^ ^]^ The structure of Au nanoparticles is diverse, and different morphologies (such as spheres, polyhedrons, core‐shell, and satellites) can be formed by regulating chemical reactions; their sizes usually range from 2 nm to 100 nm.^[^ ^254^ ^]^ Au nanoparticles are widely utilized in the biomedical field for the following primary reasons: 1) Au nanoparticles have good chemical stability and biocompatibility under physiological conditions^[^ ^254^ ^]^; 2) Au nanoparticles are easily modified by thiol compounds and can also load drug molecules through physical adsorption mediated by Coulomb and van der Waals forces;^[^ ^255^, ^256^ ^]^ 3) Au nanoparticles have a unique electronic structure, and their surface plasmon resonance effect can induce photothermal effects and can also enable label‐free detection of biomolecules.^[^ ^257^, ^258^ ^]^ Ag nanoparticles have properties similar to those of Au nanoparticles; however, they have significant bacteriostatic and bactericidal effects and can be used for the antibacterial treatment of drug‐resistant bacteria.^[^ ^259^ ^]^

#### 4.1.2 Carbon Dots

Carbon dots (CDs), also known as carbon nanodots or carbon quantum dots, consist of discrete spherical carbon nanoparticles with a size of less than 10 nm (Figure 2).^[^ ^260^ ^]^ Compared to other kinds of nanomaterials, CDs have the following advantages: 1) compared with other types of carbon nanomaterials such as carbon nanotubes and graphene, CDs are simpler to synthesize and their scale is more suitable for realizing CNS drug delivery;^[^ ^261^ ^]^ 2) compared with other inorganic nanocarriers, CDs have better biocompatibility and stability and low toxicity due to their carbon‐based composition;^[^ ^262^ ^]^ 3) compared with other organic nanomaterials such as liposomes, CDs possess smaller scales and unique photoluminescence properties, which can be widely used in drug tracking and cell imaging;^[^ ^263^ ^]^ and 4) the remnants of TCM subsequent to the process of decoction are replete with substantial quantities of cellulose, lignin, and polysaccharides, constituting valuable precursor materials for the synthesis of CDs. This utilization significantly enhances resource efficiency while concurrently mitigating environmental pollution.^[^ ^264^ ^]^

#### 4.1.3 Mesoporous Silica Nanoparticles

MSNs are prevalent nanosilicon materials with uniform narrow pore size distributions and ordered pore structures. MSNs have the following advantages owing to their structural peculiarities: 1) high specific surface area (>1000 m^2^ g^−1^), 2) adjustable pore size, 3) good biocompatibility, and 4) easy functionalization (Figure 2).^[^ ^265^, ^266^, ^267^, ^268^ ^]^ Furthermore, MSNs have been shown to have the ability to penetrate the BBB through simple surface modification (surface charge and size) and are often used to control drug release and targeted therapy in brain tumors.^[^ ^269^ ^]^

Common toxic side effects of inorganic nanocarriers include inflammation and oxidative stress. For instance, oral administration of Ag nanoparticles or silica nanocarriers can cause local inflammation,^[^ ^270^, ^271^ ^]^ whereas titanium dioxide nanocarriers interacting with the human body can stimulate the generation of ROS.^[^ ^272^ ^]^ Furthermore, research suggests that the prolonged use of inorganic nanocarriers may lead to potential adverse risks, such as the selective accumulation of Au NPs at vascular sites, exacerbating the risk of cardiovascular diseases.^[^ ^273^ ^]^ The toxic mechanisms of inorganic nanocarriers are typically associated with their size and structure.^[^ ^242^ ^]^ With increasing research on nanocarriers and technological advancements, it is possible to reduce the potential toxic effects by judiciously designing the size, structure, and surface properties of inorganic nanocarriers. This paves the way for safer and more controlled utilization of nanocarriers in TCM and their active components for disease treatment.

### 4.2 Organic Nanocarriers

**Figure 3:**
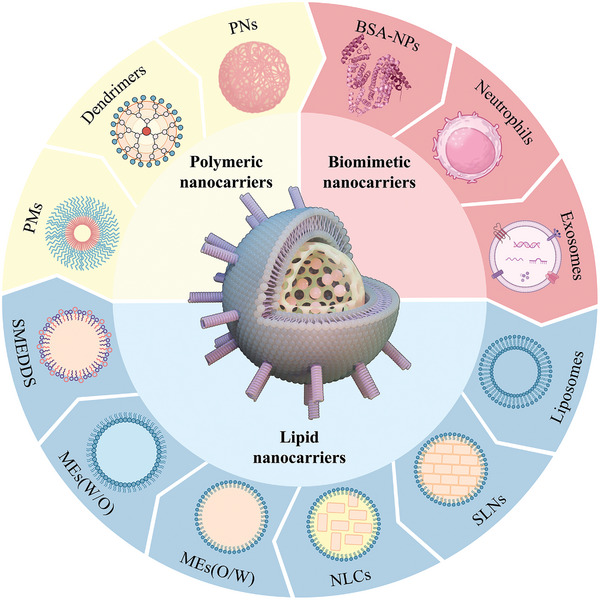
Schematic diagram of various organic nanocarriers. In the center is a hybrid nanocarrier with a drug loaded in the core, surrounded by structural diagrams of polymeric nanocarriers, biomimetic nanocarriers, and lipid nanocarriers.

Nanomaterials based on organic molecules exhibit excellent biocompatibility, extended half‐lives, high drug loading rates, considerable flexibility, and the ability to incorporate various pharmaceuticals, making them the predominant drug delivery vehicles (**Figure** **3**).

#### 4.2.1 Lipid Nanocarriers

##### Liposomes

Liposomes are spherical vesicles enclosed in one or more phospholipid bilayers. The polar groups of phospholipids orient toward the inner and outer aqueous phases,^[^ ^274^ ^]^ facilitating the encapsulation of lipophilic substances within the lipid membrane and the entrapment of hydrophilic substances within the aqueous core, respectively.^[^ ^275^ ^]^ Studies have proposed that liposomes exhibit good biocompatibility and biodegradability. They exhibit potential as active carriers with the following favorable properties: 1) improving solubility and stability;^[^ ^276^ ^]^ 2) controlling the release and increasing the survival time of the drug in vivo;^[^ ^277^, ^278^ ^]^ 3) increasing brain targeting;^[^ ^279^ ^]^ 4) enhancing bioavailability;^[^ ^280^ ^]^ and 5) realizing the co‐delivery of TCM.^[^ ^281^ ^]^

##### Lipid Nanoparticles

Solid lipid nanoparticles (SLNs) are a next‐generation nanoparticle delivery system composed of surfactants as stabilizers and solid physiological lipids (such as triglycerides, lipid acids, steroids, and glycerides) as lipid cores that encapsulate or embed drugs.^[^ ^282^ ^]^ SLNs and liposomes share a considerable degree of similarity.^[^ ^283^ ^]^ However, in comparison to liposomes, SLNs exhibit distinct advantages, namely 1) a substantially larger specific surface area and heightened drug‐carrying capacity; 2) a diminished toxicity profile and heightened biological safety; and 3) the facilitation of surface modifications, enabling the impartation of distinctive features, such as enhanced adhesion or targeted drug delivery.^[^ ^284^, ^285^ ^]^

However, despite ensuring stability, the crystalline perfection of SLNs also enforces constraints on drug incorporation within a limited space, thus challenging drug release and limiting drug‐loading capacity.^[^ ^286^, ^287^, ^288^ ^]^

In response to these challenges, nanostructured lipid carriers (NLCs) have emerged as a progressive advancement in lipid nanoparticle technology, building on the groundwork established by SLNs.^[^ ^234^ ^]^ NLCs are composed of liquid and solid lipid hybrids with irregular crystalline structures.^[^ ^283^ ^]^ Different lipid molecules are mixed to form more matrix defects to accommodate more drug molecules, thereby reducing drug excretion during storage and improving drug‐carrying capacity.^[^ ^289^ ^]^ Consequently, NLCs can deliver multiple components synergistically, thereby fulfilling multifaceted and versatile functions in tackling complex pathological conditions.

#### 4.2.2 Emulsions

##### Microemulsions

Microemulsions consist of oil, water, surfactant, and cosurfactant that spontaneously form transparent or semitransparent systems with uniform optical properties and thermodynamic stability. Microemulsions are generally spherical and very small, typically between 10 and 100 nm, and can be divided into water‐in‐oil (W/O) and oil‐in‐water (O/W), depending on the dispersed phase.^[^ ^290^, ^291^, ^292^ ^]^ As exemplary nanocarriers, microemulsions exhibit the following characteristics: 1) a straightforward preparation process, wherein the initial components can spontaneously assemble upon mixing; 2) as a thermodynamically stable system, microemulsions are easier to process, sterilize, and store; 3) microemulsions can improve the solubility of poorly soluble and lipophilic drugs and control their sustained release; and 4) microemulsions can also improve the stability, permeability, and bioavailability of drugs and increase their targeting capabilities.^[^ ^234^, ^293^, ^294^, ^295^ ^]^

##### Self‐Microemulsifying Drug‐Delivery System

The self‐microemulsifying drug delivery system (SMEDDS) is a solid or liquid concoction comprising an oil, surfactant, and cosurfactant. After entering the body, it quickly mixes with water to form a microemulsion with a droplet size of <100 nm.^[^ ^234^, ^296^ ^]^ While SMEDDS significantly improves drug solubility and bioavailability,^[^ ^296^, ^297^, ^298^ ^]^ it also has the following outstanding advantages: 1) it has high stability, can be easily manufactured, and can be produced on a large‐scale;^[^ ^234^ ^]^ 2) the oil component in SMEDDS can promote the absorption of drugs through the intestinal lymphatic system and reduce the first‐pass effect of oral drugs;^[^ ^234^ ^]^ 3) some surfactants have the function of inhibiting drug efflux, such as polyoxyethylene castor oil, polysorbate 80, and polyoxyethylene hydrogenated castor oil 40;^[^ ^299^ ^]^ and 4) targeted delivery of drugs and active ingredients can be achieved by modifying SMEDDS with ligands.^[^ ^300^ ^]^

#### 4.2.3 Polymer Nanocarriers

##### Polymer Micelles

Polymer micelles (PMs) are colloidal dispersions that originate from the self‐assembly of amphiphilic block copolymers in aqueous environments and comprise outer hydrophilic and inner hydrophobic segments.^[^ ^301^, ^302^, ^303^ ^]^ PMs have hydrophilic segments on their surface that contact the surrounding solvent and have certain passive targeting capabilities based on the enhanced permeability and retention effect. In addition, PMs can be endowed with photosensitivity, thermal sensitivity, pH sensitivity, and active‐targeting capabilities through different ligand modifications.^[^ ^304^, ^305^, ^306^, ^307^ ^]^ The hydrophobic core inside the PM can be loaded with a lipophilic drug, protecting the drug from degradation and enhancing the solubility of the drug.^[^ ^308^ ^]^ PMs have been used to treat CNS damage owing to their good stability, high drug‐loading capacity, controlled release ability, and easy surface functionalization.^[^ ^309^ ^]^

##### Polymer Nanoparticles

Polymeric nanoparticles (PNs) are nanoscale, solid, and spherical entities derived from either synthetic or naturally biodegradable polymeric materials through monomer polymerization, polymerization dispersion, or spontaneous assembly of amphiphilic polymers.^[^ ^301^, ^310^ ^]^ PMs and PNs have distinct drug‐loading modalities because of their different core structures. PMs excel at solubilizing hydrophobic drugs within their hydrophobic cores, thereby enhancing drug solubility. In contrast, PNs offer a broader range of applications capable of loading or surface adsorption of both hydrophilic and hydrophobic compounds within their solid core.^[^ ^301^ ^]^ Both PMs and PNs have similar functions; however, PNs possess the added advantage of natural biodegradability, allowing for their elimination by the body and reducing drug toxicity.^[^ ^311^, ^312^ ^]^ Moreover, functionalized polymer‐drug conjugates can be readily prepared via surface modification (e.g., antibodies, surfactants, and membrane‐penetrating peptides) to adjust the zeta potential, slow drug release, and mediate the target function.^[^ ^313^ ^]^ The common PNs approved by the FDA include polycaprolactone, polylactic acid (PLA), polyglycolic acid, polylactic acid‐co‐glycolic acid (PLGA), and chitosan, which can be used to diagnose and treat CNS damage‐related diseases.^[^ ^314^, ^315^, ^316^ ^]^

##### Dendrimers

Dendrimers are polymers characterized by dendritic 3D architectures that develop through iterative growth around the central cores, branching units, and outer active functional groups. The outstanding characteristics of dendrimers are their perfect structures and high symmetry.^[^ ^234^, ^301^ ^]^ These properties are consistent with those observed for other types of PNs.^[^ ^317^ ^]^ Additionally, dendrimers can encase drugs in the hollow cavity of the polymer skeleton, and the functional groups situated on the surface of the dendrimers can be chemically modified through conjugation, thereby enabling the attainment of specific functionalities, such as targeted interactions with specific tissues or organs.^[^ ^318^, ^319^, ^320^ ^]^ Common dendrimers are poly(amidoamine), polypropyleneimine, poly(amidoamine)organosilicon, and glycodendrimers.^[^ ^234^ ^]^

### 4.3 Biomimetic Nanocarriers

With ongoing advancements in nano‐preparation technologies, various novel nanomaterials have been developed. However, synthetic nanocarriers often have issues such as residual organic solvents, compromised stability, and potential biosafety hazards. The development of biomimetic carriers that emulate endogenous nanostructures is a viable strategy to address these challenges. Common biomimetic nanocarriers include proteins, cells, exosomes, and cell membrane vesicles, and the advantage of using endogenous substances as carriers is that they have better biocompatibility, safety, and immune evasion functions. In addition, proteins or small molecules on the surface of biomimetic carriers are also beneficial for the specific delivery of drugs to the target sites.^[^ ^121^ ^]^

Albumin, an abundant plasma protein synthesized by the liver, is primarily responsible for transporting fatty acids and hormones in the body and maintaining osmotic pressure in the blood.^[^ ^321^ ^]^ Albumin, a plasma protein abundantly synthesized by the liver, plays a primary role in transporting fatty acids and hormones and maintains osmotic pressure in the blood.^[^ ^321^ ^]^ Albumin has a variety of opportunities for surface modification and interaction with different nanoparticles owing to the presence of charged functional groups, including carboxylic and amino groups.^[^ ^322^ ^]^ Albumin nanoparticles are advantageous because of their nontoxicity, nonimmunogenicity, excellent degradability, biocompatibility, cost‐effectiveness, manufacturing simplicity, and ease of modification.^[^ ^323^, ^324^, ^325^ ^]^

Neutrophils are immune cells that can cross the BBB.^[^ ^326^ ^]^ In addition, it has been found that during the treatment of some brain diseases, such as surgical removal of gliomas, the inflammatory factors released after surgery can induce neutrophils to penetrate the BBB into the brain to reach the injury site.^[^ ^327^ ^]^ Thus, neutrophils can be used as nanocarriers to enhance the brain targeting of drugs using signals from inflammatory responses in CNS diseases.

Exosomes facilitate intercellular communication by delivering functional molecules to cells, both locally and remotely. They are 30–100 nm in diameter and exhibit a lipid bilayer structure.^[^ ^328^, ^329^ ^]^ Exosomes mediate cell‐to‐cell communication by transporting functional cargo.^[^ ^330^ ^]^ Additionally, exosomes have the advantages of low toxicity, modifiability, high biocompatibility, and specific targeting.^[^ ^331^, ^332^, ^333^ ^]^ Based on these properties, exosomes have gained wide attention and application as a natural tool for drug delivery in the field of TCM treatment of CNS diseases.^[^ ^334^, ^335^ ^]^ However, contemporary research has yet to fully elucidate the mechanisms underlying exosome action, with significant challenges remaining in their isolation and subsequent storage.^[^ ^336^ ^]^ However, this does not hinder exosomes from being considered a new type of nano‐TCM delivery carrier with broad application prospects.

### 4.4 Hybrid Nanocarriers

Each form of nanocarrier has specific advantages and functions, as well as disadvantages. For example, liposomes have problems such as poor stability and easy drug leakage, some PNs have a certain degree of cytotoxicity, and although dendrimers have a perfectly symmetrical structure, their processing costs and preparation difficulties are still very high.^[^ ^301^, ^337^ ^]^ To maximize the advantages of nanocarriers and compensate for the defects, hybrid nanocarriers formed by the combination of two or more organic/inorganic nanocarriers have been more promising drug delivery strategies in recent years. Typically, hybrid nanocarriers exhibit a core‐shell structure that arises spontaneously from amphiphilic block copolymers. The functional superposition of different types of nanocarriers in the composition gives the hybrid nanocarrier a higher drug loading rate, stability, and modifiability, which is more beneficial for the development of BBB brain‐targeted drug delivery systems (Figure 3).^[^ ^234^ ^]^

## 5 Delivery Strategy of Nano‐TCM across the BBB

**Figure 4:**
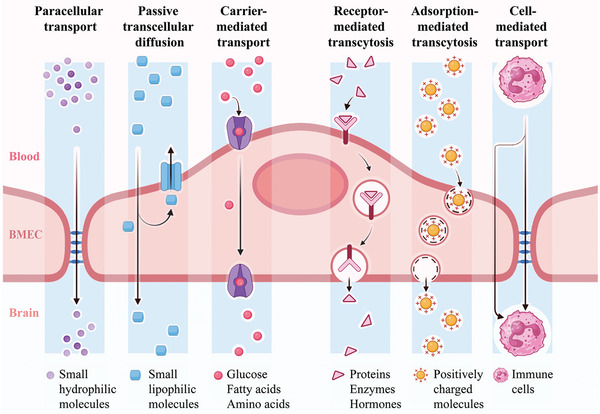
Schematic diagram of transport pathways across the blood‐brain barrier. Created with BioRender.com.

Numerous transport mechanisms exist for the delivery of nano‐TCMs to the brain via BBB targeting and are primarily classified into passive and active targeting modalities. Passive targeting typically involves substances with lower molecular weights traversing the BBB into the brain through paracellular transport or passive transcellular diffusion, whereas most other substances usually cross the BBB into the brain via active targeting, such as carrier‐mediated transport (CMT), receptor‐mediated transcytosis (RMT), adsorption‐mediated transcytosis (AMT), and cell‐mediated transport (**Figure** **4**). In addition to the aforementioned administration strategy for direct trans‐BBB transport into the brain, nano‐TCMs can also be transported to the brain by bypassing the BBB via nasal delivery. Furthermore, nanotechnology can enhance BBB permeability using physical in vitro devices, enabling more efficient targeting of TCM components or active ingredients to the brain; for example, new strategies for brain delivery, such as external magnetic field, photothermal effects, and focused ultrasound, can be achieved.

### 5.1 Passive Targeting Drug Delivery

#### 5.1.1 Paracellular Transport

Paracellular transport refers to the transport of molecules between adjacent cells. In the BBB, tight junctions between BMECs result in very small intercellular voids (<1 nm),^[^ ^338^ ^]^ suggesting that under normal physiological conditions, it is almost impossible for a drug to cross the BBB via paracellular transport. Even during pathological changes in severe CNS damage‐related diseases, the enhanced permeability of tight junctions only allows the passage of molecules <20 nm.^[^ ^339^ ^]^ Therefore, whether the integrity of the BBB is normal or compromised, the chances of a drug passing effectively through it to enter the brain are very low.

Research indicates that leveraging the unique properties of specific herbs can facilitate drugs bypassing paracellular transport barriers to penetrate the BBB and access the brain. Borneol, a plant‐based TCM categorized as a ‘Guide’ within the ‘Monarch, Minister, Assistant, and Guide’ therapeutic framework, is known for its aromatic awakening effects, and can deliver herbal medicines to the upper organs of the human body, especially to the brain.^[^ ^340^ ^]^ Additionally, recent studies have found that borneol can improve BBB permeability by regulating the expression of claudins and occludins in the tight junctions between BMECs to loosen tight junctions.^[^ ^341^, ^342^ ^]^ As a result, borneol is commonly utilized to modify nanocarriers, thereby improving their brain‐targeting efficacy and enhancing BBB permeability. Song et al. improved BBB permeability and brain targeting using borneol‐modified SLNs.^[^ ^343^ ^]^ Wang et al. improved drug delivery to the brain via intranasal administration of borneol‐modified puerarin flavone SLNs.^[^ ^344^ ^]^ Borneol is often used in conjunction with the treatment of various conditions associated with CNS impairment, such as stroke, cerebral ischemia, brain cancer, AD, PD, altered consciousness, coma, and meningitis.^[^ ^345^ ^]^ Meng et al. synthesized borneol‐modified doxorubicin polymer micelles for GBM treatment to facilitate drug penetration into the brain via the BBB.^[^ ^346^ ^]^ Ding et al. prepared liposomes loaded with borneol and angelica polysaccharides for the treatment of CIRI.^[^ ^347^ ^]^

#### 5.1.2 Passive Transcellular Diffusion

Given that the BBB comprises an anisotropic lipid bilayer at the molecular level,^[^ ^348^ ^]^ certain small lipophilic molecules, including oxygen, carbon dioxide, and alcohol, can penetrate the brain via passive transcellular diffusion.^[^ ^30^ ^]^ Importantly, brain access via this method is contingent on stringent criteria, including high lipid solubility, a molecular weight of less than 400–500 Da, and fewer than nine hydrogen bonds.^[^ ^10^ ^]^ Moreover, lipophilic molecules that infiltrate the brain via passive diffusion are cleared by efflux pumps and are influenced by efflux transporters.^[^ ^313^ ^]^ Therefore, TCM has rather limited access to the brain through this pathway.

Contemporary technological approaches can enhance the lipophilicity of drug molecules through lipid modifications, including cyclization, acylation, halogenation, esterification, and methylation,^[^ ^9^ ^]^ and can also facilitate transcellular lipophilic transport by means of nanolipid carriers. Nanotechnological modification of the molecular weight and lipophilicity of TCM components can significantly boost brain‐targeting efficiency via passive transcellular diffusion. Sharma et al. prepared a rutin (a flavonoid compound) nanoemulsion delivery system for transcellular lipophilic transport into the brain to improve free radical‐induced oxidative stress PD pathology.^[^ ^349^ ^]^ Xu et al. employed a modified direct hydration methodology to encapsulate the anticancer agent PTXL within a core‐satellite architecture consisting of polyethylene glycol (PEG)‐b‐poly(ε‐caprolactone) for the treatment of glioma. Notably, it downregulated the expression of the drug transporter P‐glycoprotein during passive transcellular diffusion, thus overcoming drug resistance.^[^ ^350^ ^]^

### 5.2 Active Targeting Drug Delivery

#### 5.2.1 Carrier‐Mediated Transport

BMECs possess a multitude of specific transporters on their surface that are designed to recognize and bind endogenous substances, including amino acids, glucose, fatty acids, carbohydrates, calcium ions, hormones, and choline, in a process known as CMT.^[^ ^351^ ^]^ In line with the CMT approach, utilizing surface modification techniques of nanocarriers can lead to the development of compounds that can be specifically recognized and bound for translocation across the BBB into the brain. For example, Li et al. coupled ginkgolide B with the unsaturated fatty acid docosahexaenoic acid and encapsulated it in liposomes for the targeted treatment of CIRI.^[^ ^352^ ^]^

Nevertheless, several factors can impede drug entry into the brain via CMT. First, CMT is concentration‐limited, and molecules are usually transported in the direction of the concentration gradient. Second, the number of transporters in CMT is limited, and conformational changes occur after the identification of endogenous substances. Third, CMT can only transport substances that are structurally similar to the endogenous ligands of the organism. Fourth, CMT transporters are widely distributed in various tissues and do not exhibit specific expression patterns in the brain.^[^ ^27^ ^]^ Therefore, CMT strategies have fewer applications in brain‐targeted delivery systems of nano‐TCMs and often require other designs.

#### 5.2.2 Receptor‐Mediated Transcytosis

RMT refers to the uptake of macromolecular substances, such as hormones, growth factors, plasma proteins, proteins, and enzymes, into the brain by specific RMT on the surface of BMECs.^[^ ^353^, ^354^ ^]^ RMT occurs primarily through clathrin‐mediated processes. When a macromolecular ligand binds to specific receptors, the resultant ligand‐receptor complex aggregates in clathrin‐coated pits on the cell membrane, and the membrane invaginates to form vesicles. Upon entry into cells, these vesicles give rise to early endosomes, within which the complexes dissociate in an acidic environment, allowing ligands to either continue transcytosis into the brain or be degraded by lysosomes.^[^ ^313^ ^]^ There are many receptors on the BBB involved in RMT, including lactoferrin, transferrin, insulin, endothelial growth factor, amino acid, and tumor necrosis factor receptors.^[^ ^121^, ^313^ ^]^ Additionally, the upregulation of specific receptors occurs during the pathological processes of certain CNS damage‐related diseases. Capitalizing on this property, nanocarriers can be surface‐functionalized using ligands corresponding to upregulated receptors to target drug‐loaded nanoparticles via RMT across the BBB into the brain.^[^ ^355^ ^]^ For example, Pavlov et al. encapsulated the antitumor drug PTXL within cerasomes, which were modified with the nonionic surfactant Tween 80, to facilitate targeted treatment of GBM. It merits attention that Tween 80 binds to apolipoproteins in the bloodstream and crosses the BBB via a receptor‐mediated route.^[^ ^356^ ^]^ Qi et al. designed liposomes loaded with docetaxel (DTXL), which were dual‐modified with a lactoferrin receptor and muscone, to cross the BBB through a receptor‐mediated mechanism, ultimately bolstering the therapeutic effectiveness against glioma.^[^ ^230^ ^]^

RMT is independent of the concentration gradient and exhibits better specificity and brain targeting than CMT. However, competition with endogenous substances for a limited number of receptors on the surface of BMECs is also a problem. In addition, there is a potential for the drug to be degraded by lysosomes during its passage via RMT. Consequently, advancing drug delivery across the BBB through RMT necessitates the synthesis of novel materials that demonstrate enhanced structural stability and evade lysosomal degradation.

#### 5.2.3 Adsorption‐Mediated Transcytosis

AMT is the process by which a positively charged substance comes in contact with a negatively charged plasma membrane surface, producing an electrostatic effect that initiates endocytosis, thereby facilitating the passage of the substance through the BBB into the brain.^[^ ^357^ ^]^ Similarly, AMT strategies can be implemented by the functional modification of nanocarriers. Drug‐loaded NPs can be cationically delivered to the brain via AMT.^[^ ^358^ ^]^ For example, Kamalinia et al. used cationized human serum albumin to deliver drugs into the brain via AMT, effectively preventing apoptotic cell death in AD and ameliorating Aβ‐induced learning deficits.^[^ ^358^ ^]^ On the other hand, drug‐loaded NPs can bind to positively charged groups to enhance endocytosis, thereby improving brain targeting. For instance, cell‐penetrating peptides (CPPs), typically composed of 5–30 amino acids and serving as targeting vectors, are usually positively charged and amphiphilic, enabling them to penetrate the plasma membrane and transport molecules into cells;^[^ ^359^ ^]^ these CPPs are frequently utilized to convey nano‐TCM into the brain via the AMT strategy. Building upon this premise, Li et al. utilized the CPP dNP2 to modify liposomes for the targeted delivery of PTXL to gliomas in the brain.^[^ ^359^ ^]^ Additionally, Caban et al. utilized positively charged chitosan‐modified PLGA NPs for the co‐delivery of PTXL and flurbiprofen, achieving precise targeting of the glioma tissue.^[^ ^360^ ^]^

AMT has a lower affinity than RMT, lacks specificity, can occur in blood vessels at other sites,^[^ ^361^ ^]^ and has some toxic effects at large doses.^[^ ^362^ ^]^ However, the vesicles formed during AMT have a larger volume, indicating that they can accommodate a drug with a larger molecular weight.^[^ ^9^ ^]^ Furthermore, nanoparticles can escape lysosomal degradation when crossing the BBB via the AMT pathway^[^ ^27^ ^]^ and can serve as an auxiliary strategy for RMT. For example, Liu et al. enhanced the bioavailability of baicalin and its permeability across the BBB using PEGylated cationic solid lipid nanoparticles loaded with a baicalin surface coupled with an OX26 antibody (a transferrin receptor monoclonal antibody)^[^ ^363^ ^]^ that effectively relieved neuronal damage during CIRI by modulating amino acid levels in the CSF after entering the brain. In conclusion, AMT is a promising strategy for the targeted brain delivery of nano‐TCM.

#### 5.2.4 Cell‐Mediated Transport

In the context of this review, cell‐mediated transport pertains to the pathological processes in CNS diseases wherein cells such as monocytes, neutrophils, and macrophages, which originate from mononuclear cells, are extensively recruited and transported into the brain parenchyma via circulation during the inflammatory phase.^[^ ^355^ ^]^

The traditional drug delivery system design avoids uptake by immune cells as much as possible, while the contrary is true for the cell‐mediated transport strategy, wherein drug molecules or drug‐loaded nanoparticles are embedded into cells with a specific tendency to become a “Trojan horse” that crosses the BBB into the brain and releases the drug at the site of injury. Given the improved drug delivery loading, targeting, and survival of endogenous cells, this bio‐nanotechnology is less cytotoxic and immunogenic than artificially prepared materials and represents a safer and more precise treatment strategy for targeting CNS diseases.^[^ ^364^ ^]^ For instance, Wang et al. devised a “Trojan horse” in which PLGA nanoparticles loaded with PTXL were inserted into mesenchymal stem cells, taking advantage of their tropism and low immunity to tumor cells.^[^ ^365^ ^]^ In addition, Du et al. capitalized on the exceptional BBB penetration capability of engineered microglia (BV2 cells), utilizing them as a conveyance system to transport PTXL‐loaded liposomes, thereby efficiently delivering the drug to glioma cells via characteristic extracellular vesicles and tunneling nanotube transport mechanisms.^[^ ^366^ ^]^

However, cell‐mediated transport also has some problems that need to be addressed, such as premature release of loaded drugs, clearance mechanisms of immune cells, and off‐target dispersion of drugs in the absence of inflammatory stimuli.^[^ ^353^, ^364^ ^]^ Nonetheless, cell‐mediated transport strategies have opened new pathways for the development of nano‐TCM delivery systems.

### 5.3 Intranasal Drug Delivery

**Figure 5:**
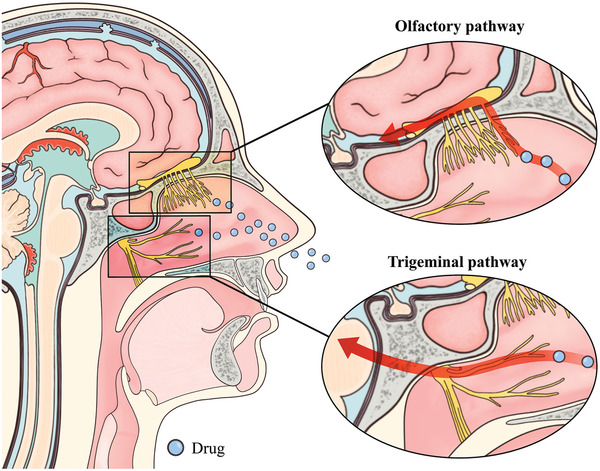
Schematic diagram of intranasal drug delivery. Drugs in the nasal cavity bypass the blood‐brain barrier and enter the brain via olfactory or trigeminal pathway.

In the mid‐19th century, the speculation of a transmission pathway between the brain and nasal cavity arose from the observation that a dye injected into the subarachnoid space appeared in the deep cervical lymph nodes, a method that was first applied in 1989 to treat CNS diseases.^[^ ^367^ ^]^ Unlike the delivery approach described above for the direct crossing of the BBB in active or passive targeting, nasal delivery bypasses the BBB into the CNS along the olfactory or trigeminal pathways (**Figure** **5**).^[^ ^368^ ^]^ First, nasal administration is a noninvasive route that offers rapid absorption and swift onset of action compared to conventional intravenous administration.^[^ ^369^ ^]^ Second, unlike oral administration, nasal administration avoids the first‐pass effect of the liver,^[^ ^370^ ^]^ which is an excellent option for TCM with generally lower oral bioavailability. Furthermore, the incorporation of viscous nanomaterials, such as hydrogels, can prolong the adherence of drugs to the nasal mucosa, facilitating sustained drug release. This mode of administration not only improves the bioavailability of the drug but also contributes to patient compliance and comfort.^[^ ^371^ ^]^ For instance, Huang et al. developed a drug delivery system loaded with the volatile oil of Chaxiong microemulsion thermosensitive in situ gel by nasal administration for the treatment of ischemic stroke.^[^ ^371^ ^]^ Traditional volatile oils derived from tea lovage rhizomes are characterized by low water solubility, high volatility, and poor oral bioavailability. By prolonging the retention time of the tea lovage volatile oil in the nasal mucosa, the in situ gel further promoted the dissolution and permeation of the medicament via the nasal‐brain route and effectively improved its bioavailability. Similarly, Fonseca et al. used in situ gelling liquid crystals in the nasal cavity as a delivery system for resveratrol to mitigate the neuroinflammation and memory deficits associated with AD.^[^ ^372^ ^]^

However, from a practical standpoint, there is a risk that the drug may be inadvertently inhaled into the lungs or ingested into the gastrointestinal tract.^[^ ^373^ ^]^

Prolonged drug administration may exert toxic effects and induce side effects on the nasal mucosa and ciliary structures within the nasal cavity.^[^ ^374^ ^]^

### 5.4 Physical Targeting Drug Delivery

Traditional drug delivery strategies rely on physiological pathways within the body. In recent years, physical methods, including magnetic fields, photothermal effects, or focused ultrasound applied externally, have demonstrated significant potential in enhancing the BBB permeability,^[^ ^375^ ^]^ as summarized in **Table** **1**.

**Table 1:** Summary of physical targeting drug delivery.

Nano‐TCM	Components responsive to physical signals	physical signals	Mechanism	Performance
Res‐lips@Fe3O4^[^ ^378^ ^]^	Fe_3_O_4_ NPs	Magnetic field	Magnetic targeting	With high drug‐loading capacity, stability, and strong magnetic targeting. Facilitate drug penetration through the BBB under an external magnetic field, increasing drug concentration at the target site, and enhancing therapeutic efficacy.
MSNs‐AuNRs@QCT^[^ ^386^ ^]^	AuNRs	NIR‐II light	Photothermal effect	Exhibits outstanding stability, biocompatibility, and high BBB permeability. Significantly reduces neuronal damage, improving neurofunctional disorders.
CPC NPs^[^ ^389^ ^]^	microbubbles	Focused ultrasound	cavitation effect	Enables local, non‐invasive opening of the BBB, achieving targeted delivery into the brain. Possesses high drug‐loading capacity and stability, effectively improving neural behavioral deficits.

**Figure 6:**
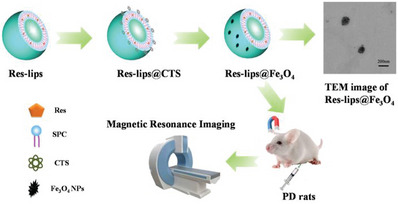
Preparation Flowchart of Res‐lips@ Fe3O4 as a Novel Diagnostic and Therapeutic Platform for PD. Reproduced with permission.^[^ ^378^ ^]^ Copyright 2018, American Chemical Society.

Magnetic nanoparticles have been extensively studied and utilized for the localization, imaging, and treatment of CNS diseases because of their distinctive magnetic properties. Iron oxide nanoparticles (IONPs) are employed in magnetic resonance imaging (MRI) and magnetically targeted drug delivery. The application of a magnetic field in combination with IONPs disrupts the tight junctions of the BBB, facilitating the seamless passage of drug‐laden IONPs into the brain.^[^ ^376^, ^377^ ^]^ For instance, Wang et al. developed a magnetically targeted drug delivery system using Fe_3_O_4_‐modified resveratrol liposomes for PD (**Figure** **6**).^[^ ^378^ ^]^ When used in conjunction with an external magnetic field, the exhibited magnetic responsiveness enhanced BBB permeability and MRI diagnostic capabilities. This demonstrates the viability of the magnetic targeting approach and offers a novel concept for treating CNS diseases using nano‐TCMs that cross the BBB. Notably, unmodified magnetic nanoparticles not only tend to aggregate and block capillaries but also exhibit cytotoxicity.^[^ ^379^, ^380^ ^]^ However, in recent years, an increasing variety of inorganic and organic coating materials have been developed to prevent the agglomeration of magnetic nanoparticles, enhance their stability, and reduce their toxicity.^[^ ^381^, ^382^, ^383^ ^]^

**Figure 7:**
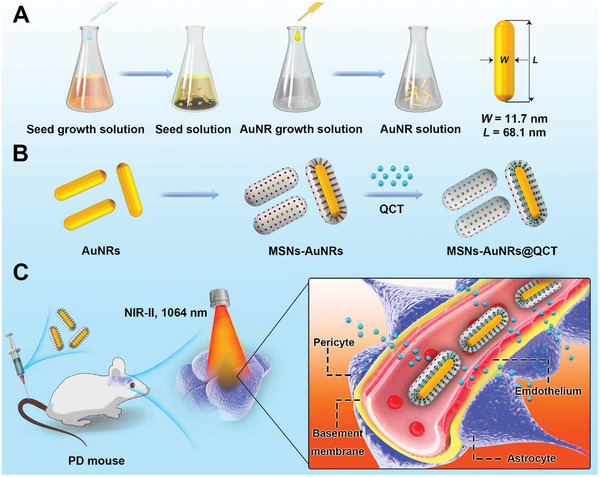
Illustration of the fabrication process for MSNs‐AuNRs@ QCT and a schematic depiction of its mechanism for crossing the BBB under NIR‐II irradiation. Reproduced with permission.^[^ ^386^ ^]^ Copyright 2020, American Chemical Society.

Near‐infrared (NIR) light, which can penetrate tissues, is applicable in biological settings.^[^ ^384^ ^]^ NIR irradiation can penetrate up to 2.4 mm beneath the scalp and skull, creating a ‘biological window’ that serves as a conduit for drug delivery.^[^ ^385^ ^]^ A photothermal effect‐driven brain‐targeting nano‐TCM delivery system can be achieved by amalgamating various nanomaterials with photothermal capabilities to transport TCM. Augmenting BBB permeability through photothermal effects has broad applicability in the treatment of CNS diseases. Liu et al. engineered a nanocomposite featuring an egg yolk‐like shell structure that encapsulated mesoporous silica‐coated gold nanorods loaded with QCT for PD therapy (**Figure** **7**).^[^ ^386^ ^]^ Upon NIR‐II irradiation, gold nanorods modulated NIR‐induced surface plasmon resonance,^[^ ^387^ ^]^ causing irradiated electrons to return to the ground state and emit substantial heat. This photothermal effect increased BBB permeability, significantly enhanced the delivery efficiency of QCT to the brain, and markedly reduced neuronal damage and associated dysfunction in PD models. In terms of safety, the interaction between NIR‐II light and biological tissues is extremely weak, with minimal phototoxicity and excellent tolerance. However, because most nanomaterials used in conjunction with NIR‐II are derived from the modifications of materials previously associated with NIR‐I, their light absorption capacity and biocompatibility have not yet reached optimal levels. Careful consideration is required for the selection and design of nanomaterials for various applications, and strict control of the toxic side effects associated with nanomaterials is essential.^[^ ^384^ ^]^ However, advancements in laser technology suggest that the use of this photothermal effect in nano‐TCMs has a vast potential for application.

**Figure 8:**
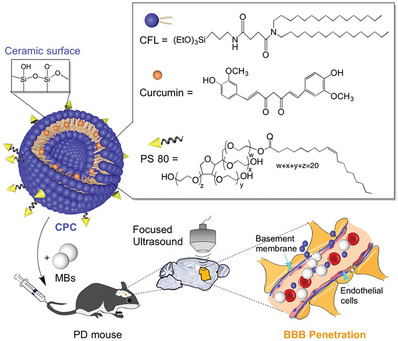
Using FUS technology for noninvasive targeted delivery of CPC nanoparticles across the BBB for the treatment of PD. Reproduced with permission.^[^ ^389^ ^]^ Copyright 2018, Ivy Spring International Publisher.

Focused ultrasound (FUS) directs sound wave energy to specific areas within the body such as the BBB to reversibly open tight junctions between endothelial cells. This is achieved by using microbubble contrast agents that generate oscillatory and cavitation effects, thereby creating a brief period during which drugs can be more effectively targeted to the brain.^[^ ^388^ ^]^ Zhang et al. formulated a delivery system that encapsulated CUR in polysorbate 80‐coated cerasomes (CPC) and used ultrasound‐targeted microbubble destruction to augment PD treatment (**Figure** **8**).^[^ ^389^ ^]^ A notable 1.7‐fold increase in CUR accumulation was observed within the left striatum of mice compared to that in the right striatum, 6 h after the administration of CPC and microbubbles. Additionally, this combined treatment significantly improved dopamine levels and reduced behavioral deficits in PD mice within a mere two weeks. These findings underscore the efficacy of FUS‐binding microbubbles in achieving noninvasive local disruption of the BBB, facilitating the targeted delivery of CPC nanoparticles to specific brain regions. However, the use of ultrasound‐mediated targeted therapy is not yet widespread and requires further investigation and refinement. Excessively high sound pressure may result in intense microbubble cavitation and consequent damage to adjacent tissues,^[^ ^390^ ^]^ potentially leading to adverse outcomes and brain injuries, including hemorrhage and inflammation.^[^ ^121^ ^]^ Nonetheless, there is reason to believe that with ongoing technological advancements, this innovative FUS‐based targeted therapy will inevitably emerge as a safer and more efficacious treatment modality for CNS diseases.

## 6 Application of Nano‐TCM in the Treatment of CNS disease

With the advent of diverse systems, such as inorganic nanocarriers, organic nanocarriers, and inorganic/organic hybrid nanocarriers, nano‐TCM has advanced significantly in the realm of drug delivery for CNS disease treatment. Researchers have explored various therapeutic strategies to cross or circumvent the BBB. Combined with innovative preparation methods, such as high‐pressure microfluidization, microwave technology, supercritical fluid technology, vacuum freeze‐drying, media milling, precipitation, dialysis, self‐assembly, and spray drying, TCM has been reformulated into nanoscale formulations.^[^ ^391^ ^]^ Through these modern techniques, highly effective TCM components with low BBB permeability and reduced bioavailability have been successfully targeted to the cells and tissues of the CNS (**Table** **2**). To this end, we examined the research on nano‐TCM applications in this field, categorized by specific CNS diseases.

**Table 2:** Summary of application of nano‐TCM in the treatment of CNS disease.

Diseases	Formulations	Preparation method	Size[nm]	Mechanisms	BBB permeability	Administration route	Active ingredients
AD	Carbon dots	Ultrasonication‐mediated method	3.4 ± 1.0	Passive diffusion	–	Intravenous injection	Citric acid^[^ ^261^ ^]^
AD	Sulfur nanoparticles	One‐pot method	53 ± 15	Paracellular transport	–	Intravenous injection	Quercetin^[^ ^444^ ^]^
AD	Selenium nanoparticles	Freeze‐drying method and Solvent evaporation method	<200	Receptor‐mediated transcytosis	–	Intravenous injection	Quercetin^[^ ^445^ ^]^
AD	Liposomes	Lipid injection method	–	/	–	/	Curcumin^[^ ^446^ ^]^
AD	Liposomes	Thin‐film hydration method	200	Receptor‐mediated transcytosis	2–3‐fold increase	/	Curcumin, quercetin Epigallocatechin gallate Rosmarinic acid^[^ ^398^ ^]^
AD	Liposomes	Vacuum vapor deposition method	–	Receptor‐mediated transcytosis	2‐fold increase	/	Quercetin^[^ ^399^ ^]^
AD	Liposomes	Thin‐film hydration method	158.5	Receptor‐mediated transcytosis Adsorption‐mediated transcytosis	3‐fold increase	Intravenous injection	Rosmarinic acid Curcumin Quercetin^[^ ^447^ ^]^
AD	Solid lipid nanoparticles	Micro‐emulsion method	< 200	/	4.5‐fold increase	Oral administration	Resveratrol^[^ ^448^ ^]^
AD	Solid lipid nanoparticles	Ultrasonication method and high shear homogenization method	176 ± 24	Receptor‐mediated transcytosis	4‐fold increase	/	Resveratrol Grape extract^[^ ^449^ ^]^
AD	Solid lipid nanoparticles Nanostructured lipid carriers	Sonication method and hot homogenization method	SLN: 234 ± 18 NLC: 219 ± 13	Receptor‐mediated transcytosis	–	/	Quercetin^[^ ^400^ ^]^
AD	Solid lipid nanoparticles Nanostructured lipid carriers	Sonication method and hot homogenization method	SLN: 201 ± 23 NLC: 222 ± 22	Receptor‐mediated transcytosis	1.5‐fold increase	/	Quercetin^[^ ^450^ ^]^
AD	Microemulsion	Water titration method	O/W ME:131.80 ± 13.09 W/O ME:132.52 ± 10.63	Receptor‐mediated transcytosis	–	Oral administration	Piperine^[^ ^451^ ^]^
AD	Polymeric nanoparticles	Nanoprecipitation method	145.2	Receptor‐mediated transcytosis	–	Intravenous injection	Rhynchophylline^[^ ^395^ ^]^
AD	Polymeric nanoparticles	Emulsion solvent evaporation method	200 ± 20	Receptor‐mediated transcytosis	2.1‐2.8‐fold increase	Intraperitoneal injection	Curcumin^[^ ^452^ ^]^
AD	In situ gel	Melt emulsification‐probe sonication method	132 ± 11.90	Nasal‐to‐brain	5‐fold increase	Intranasal administration	Resveratrol^[^ ^453^ ^]^
AD	In situ gel	Self‐assembly method		Nasal‐to‐brain	3.5‐fold	Intranasal administration	Resveratrol^[^ ^372^ ^]^
AD	Human serum albumin nanoparticles and Red blood cell membrane	NHS‐amino coupling reaction	<120	Cell‐mediated transport	5.6‐fold increase	Intravenous injection	Curcumin^[^ ^404^ ^]^
AD	Exosomes	Ultrasonic incubation method	150	Receptor‐mediated transcytosis	2.5‐fold increase	Intravenous injection	Quercetin^[^ ^405^ ^]^
AD	Exosomes	Co‐incubation method	117.4 ± 10.5	Receptor‐mediated transcytosis	6.5‐fold increase	Intravenous injection	Curcumin^[^ ^406^ ^]^
AD	Exosome‐like liposomes	Thin‐film hydration method	< 200	Receptor‐mediated transcytosis	–	Oral administration	Curcumin^[^ ^408^ ^]^
PD	Mesoporous silica and Gold nanorods	Incubation and self‐assembly methods	15.7×91.3	Photothermal effect	2.9‐fold increase	Intravenous injection	Quercetin^[^ ^454^ ^]^
PD	Liposomes	Chemical co‐precipitation method	155.7 ± 1.6	Paracellular transport	2‐fold increase	Intraperitoneal injection	Resveratrol^[^ ^378^ ^]^
PD	Liposomes	Thin‐film hydration method	110	Paracellular transport	1.7‐fold increase	Intravenous injection	Curcumin^[^ ^389^ ^]^
PD	Lipid nanocarrier	Modified emul‐siosonication method	92.46–95.34	Receptor‐mediated transcytosis	–	Oral administration	Quercetin^[^ ^455^ ^]^
PD	Microemulsion	High‐pressure homogenization method and spontaneous emulsification method	18 ± 0.01	Passive transcellular diffusion	1.8‐fold increase	Oral administration	Rutin^[^ ^349^ ^]^
PD	Nanoemulsion	High‐shear homogenization method	50	/	–	Oral administration	Quercetin^[^ ^456^ ^]^
PD	Polymeric nanoparticles	Flash nanoprecipitation method	70	Passive transcellular diffusion	31.86‐fold increase	Oral administration	Schisantherin A^[^ ^412^ ^]^
PD	Polymeric nanoparticles	Reprecipitation self‐assembly method	131.1	Nasal‐to‐brain	–	Intranasal administration	Curcumin analog^[^ ^410^ ^]^
PD	Neuronal cell membrane	Aqueous method	24.7	Paracellular transport	–	Intravenous injection	Quercetin^[^ ^414^ ^]^
PD	Liposomes and Natural killer cell membrane	Repeated freeze‐thaw method	82	Meningeal lymphatic vessel route	20‐fold increase (through the MLVs pathway)	Subcutaneous injection	Curcumin^[^ ^413^ ^]^
PD	Nanocrystals	Antisolvent precipitation method	83.05 ± 1.96	/	6.44‐fold increase	Oral administration	Puerarin^[^ ^457^ ^]^
PD	ZIF‐8@PB	Vacuum freeze‐drying method	107	Paracellular transport	2.67‐fold increase	Intravenous injection	Quercetin^[^ ^415^ ^]^
cerebral ischemia‐reperfusion injury	Liposomes	Film dispersion method	109.3 ± 1.68	Carrier‐mediated transport	2.2‐fold increase	Intravenous injection	Ginkgolide B^[^ ^352^ ^]^
cerebral ischemia‐reperfusion injury	Liposomes	Thin‐film hydration method	179.1	Paracellular transport	–	Intravenous injection	Borneol Angelica polysaccharide^[^ ^347^ ^]^
cerebral ischemia‐reperfusion injury	Liposomes	Reverse evaporation method	–	Paracellular transport	1.6‐fold increase	Intravenous injection	Borneol Baicalin^[^ ^421^ ^]^
cerebral ischemia‐reperfusion injury	Liposomes	Reverse evaporation method	167.1	Paracellular transport	–	Intravenous injection	Borneol Baicalin^[^ ^422^ ^]^
Cerebral ischemic stroke	Liposomes	Thin‐film ultrasonic dispersion method	128.01 ± 5.91	Paracellular transport	6.53‐fold increase	Intravenous injection	Borneol Ginkgolides^[^ ^423^ ^]^
Cerebral ischemic stroke	SMEDDS	Self‐emulsification method	151.6 ± 1.92	Paracellular transport	10.27‐fold increase	Oral administration	Borneol Puerarin^[^ ^458^ ^]^
Cerebral ischemic stroke	Polymeric nanoparticles	Self‐assembly method	104.8	/	–	Lateral ventricle injection	18β‐glycyrrhetic acid^[^ ^417^ ^]^
Intracerebral hemorrhage	Polymeric nanoparticles	Antisolvent precipitation method	127.31 ± 2.73	Receptor‐mediated transcytosis	–	Oral administration	Curcumin^[^ ^418^ ^]^
Cerebral ischemia‐reperfusion injury	Polymeric nanoparticles	–	100	/	–	Intra‐carotid artery administration	Resveratrol^[^ ^419^ ^]^
Diabetic cerebral infarction	Polymeric nanoparticles	Vacuum freeze‐drying method	89 ± 23	Receptor‐mediated transcytosis	13.53‐fold increase	Intravenous injection	Ginsenoside rg1^[^ ^420^ ^]^
Cerebral ischemia‐reperfusion injury	Polymeric nanoparticles Nanostructured lipid carriers	Low‐temperature emulsion evaporation solidification method	181.3 ± 5.6	Paracellular transport	–	Intravenous injection	Tanshinol borneol ester^[^ ^424^ ^]^
Cerebral ischemia‐reperfusion injury	Liposomes Neutrophils	Co‐incubation method	107.26 ± 0.55	Cell‐mediated transport Adsorptive‐mediated transcytosis	3‐fold increase	Intravenous injection	Puerarin^[^ ^425^ ^]^
Cerebral ischemic stroke	Microemulsion and In situ gel	Cold method and emulsion phase inversion method	21.02 ± 0.25	Nasal‐to‐brain	2.01‐fold increase	Intranasal administration	Chaxiong volatile oil^[^ ^371^ ^]^
Glioma	Carbon dots	One pot hydrothermal method	2 ± 0.5	Passive diffusion	–	Intravenous injection	Gallic acid^[^ ^459^ ^]^
Glioma	MnO2	Dialysis method and bioconjugation method	159.8 ± 4.9	Receptor‐mediated transcytosis	2.93‐fold increase	Intravenous injection	Paclitaxel^[^ ^432^ ^]^
Glioma	Iron oxide nanoparticle	Dialysis method	37 ± 3	Paracellular transport	–	Intravenous injection	Paclitaxel^[^ ^431^ ^]^
Glioma	Nanofibers	Dialysis method and electrospinning method	235 ± 95 – 410 ± 320	Paracellular transport	–	/	Paclitaxel^[^ ^460^ ^]^
Glioma	Liposomes	Ethanol sol injection method Thin‐film hydration method	300‐400 20‐200	Receptor‐mediated transcytosis	–	Intravenous injection	Paclitaxel^[^ ^356^ ^]^
Glioma	Liposomes	Thin‐film hydration method	150	Receptor‐mediated transcytosis	–	Intravenous injection	Muscone^[^ ^461^ ^]^
Glioma	Liposomes	Thin‐film hydration method	66.12 ± 0.393	Receptor‐mediated transcytosis	5‐fold increase	Intravenous injection	Paclitaxel Ginsenoside Rg3^[^ ^462^ ^]^
Glioma	Liposomes	Dialysis method and bioconjugation method	103.77 ± 0.65	Receptor‐mediated transcytosis	6.03‐fold increase	Intravenous injection	Resveratrol^[^ ^463^ ^]^
Glioma	Liposomes	Solvent evaporation method and Thin‐film hydration method	128.15 ± 1.63	Receptor‐mediated transcytosis	2‐fold increase	Intravenous injection	Paclitaxel^[^ ^464^ ^]^
Glioma	Liposomes	Thin‐film dispersion method	144.6 ± 3.1	Receptor‐mediated transcytosis	3.17‐fold increase	Intravenous injection	Muscone docetaxel^[^ ^230^ ^]^
Glioma	Liposomes	Thin‐film hydration method	181 ± 2.3	Receptor‐mediated transcytosis	–	/	Curcumin camptothecin^[^ ^465^ ^]^
Glioma	Solid lipid nanoparticles	Hot‐melt emulsification method and high‐speed homogenization method	234 ± 1.33	Receptor‐mediated transcytosis	–	Oral administration	Paclitaxel Naringenin^[^ ^466^ ^]^
Glioma	Polymer micelles	Self‐assembly method	14.95 ± 0.17	Paracellular transport	0.9‐fold increase	Intravenous injection	Borneol^[^ ^346^ ^]^
Glioma	Polymer micelles	Self‐assembly method	101.3 ± 8.9	Receptor‐mediated transcytosis	1.9‐fold increase	Intravenous injection	Paclitaxel^[^ ^467^ ^]^
Glioma	Polymer micelles	Film dispersion method	22.33 ± 0.99	Receptor‐mediated transcytosis Carrier‐mediated transport	1.8‐fold increase	Intravenous injection	Paclitaxel Artemether^[^ ^468^ ^]^
Glioma	Polymer micelles	Ultrasonic film hydration method	172.3	Receptor‐mediated transcytosis	2.46‐fold increase	Intravenous injection	Paclitaxel^[^ ^469^ ^]^
Glioma	Polymer micelles	Dialysis method	110	Receptor‐mediated transcytosis	–	Intravenous injection	Paclitaxel^[^ ^429^ ^]^
Glioma	Polyphenol nanoparticles	Dialysis method	16.3	Receptor‐mediated transcytosis	2.6‐fold increase	Intravenous injection	Quercetin^[^ ^430^ ^]^
Glioma	Polymeric nanoparticles	Sonication/evaporation method	200	/	–	Intravenous injection	Docetaxel Paclitaxel^[^ ^427^ ^]^
Glioma	Polymeric nanoparticles	Solid‐phase peptide synthesis	–	Receptor‐mediated transcytosis	4‐fold increase	Intravenous injection	Paclitaxel^[^ ^428^ ^]^
Glioma	Polymeric nanoparticles	Nanoprecipitation method	50‐230	Receptor‐mediated transcytosis	–	/	Paclitaxel^[^ ^470^ ^]^
Glioma	Polymeric nanoparticles	Direct hydration method	136.5 ± 0.44	Passive transcellular diffusion	–	intracranial injection	Paclitaxel^[^ ^350^ ^]^
Glioma	Polymeric nanoparticles	Nanoprecipitation method	<150	/	–	Intravenous injection	Paclitaxel^[^ ^471^ ^]^
Glioma	Polymeric nanoparticles	Nanoprecipitation method	150‐190	Adsorption‐mediated transcytosis	–	Intraperitoneal injection	Paclitaxel^[^ ^360^ ^]^
Glioma	Polymeric nanoparticles	Antisolvent method	216 ± 0.8	Nasal‐to‐brain	–	Intranasal administration	Paclitaxel^[^ ^472^ ^]^
Glioma	Polymeric nanoparticles	Dialysis method	30 ± 4.8	Receptor‐mediated transcytosis	–	Intraperitoneal injection	Resveratrol^[^ ^473^ ^]^
Glioma	Hepatitis B core protein‐virus‐like particles	Dialysis method	–	Receptor‐mediated transcytosis	4‐fold increase	Intravenous injection	Paclitaxel^[^ ^474^ ^]^
Glioma	Ferritin heavy chain nanocages	Disassembly/reassembly method	15.5	Receptor‐mediated transcytosis	10‐fold increase	Intravenous injection	Paclitaxel^[^ ^475^ ^]^
Glioma	Extracellular vesicles	Electroporation method and click chemistry method	122.7 ± 6.5	Receptor‐mediated transcytosis	–	Intravenous injection	Paclitaxel^[^ ^436^ ^]^
Glioma	Albumin	–	–	Paracellular transport	3.7‐fold increase	Intravenous injection	Paclitaxel^[^ ^433^ ^]^
Glioma	Albumin	–	–	Paracellular transport	3‐ to 5‐fold increase	Intravenous injection	Paclitaxel^[^ ^434^ ^]^
Glioma	Nanosuspensions and Biomimetic cancer cell membrane	Active ester method and ultrasound precipitation method	169.24	Receptor‐mediated transcytosis	7.4‐fold increase	Intravenous injection	Paclitaxel^[^ ^476^ ^]^
Glioma	Polymeric nanoparticles and Biomimetic cancer cell membrane	Dialysis method	107	Cell‐mediated transport	20‐fold increase	Intravenous injection	Paclitaxel^[^ ^435^ ^]^
Glioma	Liposomes and Microglia (BV2 cells)	Filming‐rehydration method	95–100	Cell‐mediated transport	–	Intravenous injection	Paclitaxel^[^ ^366^ ^]^
Traumatic brain injury	Carbon dots	Dialysis method	3.12 ± 0.95	Adsorptive‐mediated transcytosis	–	Intravenous injection	Semen pruni persicae Carthamus tinctorius L^[^ ^437^ ^]^
Encephalitis(Cytomegalovirus infection)	Liposomes	Microemulsion‐based method	142.5 ± 6.3	Active and passive pathways	2.12‐ to 3.18‐fold increase	Intravenous injection	Borneol^[^ ^477^ ^]^
Heat stroke	Nanowires	Hydrothermal synthesis method	–	/	–	Intraperitoneal injection	Gingko Biloba (EGb‐761) Bilobalide BN‐52021^[^ ^478^ ^]^
Spinal cord injury	Exosomes	Ultrasonic method	125 ± 12	Cell‐mediated transport	18.27‐fold increase	Intravenous injection	Berberine^[^ ^334^ ^]^
Huntington's Disease	Solid lipid nanoparticles	–	–	Receptor‐mediated transcytosis	–	Oral administration	Curcumin^[^ ^438^ ^]^

### 6.1 Alzheimer's Disease

**Figure 9:**
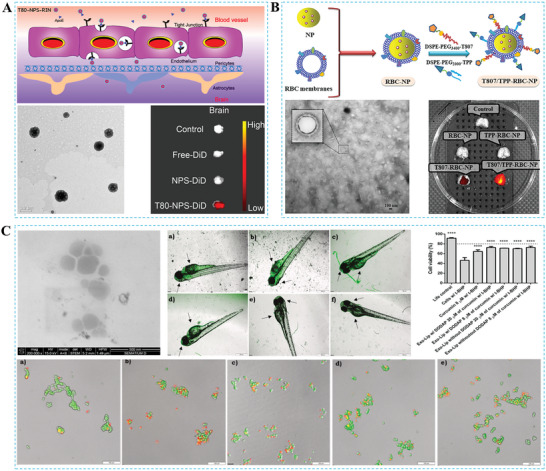
Application of nano‐TCM treatment strategies in AD. A) T80‐NPS‐RIN can efficiently traverse the blood‐brain barrier (BBB) and exert a substantial neuroprotective effect in the treatment of AD. Reproduced with permission.^[^ ^395^ ^]^ Copyright 2020, DOVE Medical Press. B) CUR‐loaded T807/TPP‐RBC‐NPs, developed utilizing bionic nanotechnology, can address AD by mitigating mitochondrial dysfunction. Reroduced with permission.^[^ ^404^ ^]^ Copyright 2020, Elsevier. C) A hybrid nanoparticle comprising exo‐liposomes facilitates the delivery of CUR to the brain for AD therapy. Reproduced with permission.^[^ ^408^ ^]^ Copyright 2021, Elsevier.

Rhynchophylline (RIN), a tetracyclic oxindole alkaloid from Uncaria species used in traditional Chinese medicine,^[^ ^392^ ^]^ can mitigate soluble Aβ‐induced hyperactivity in hippocampal neurons and is frequently employed in therapies for CNS disorders.^[^ ^393^ ^]^ However, its brain bioavailability is compromised by its low solubility, poor bioavailability, and challenges in BBB penetration.^[^ ^394^ ^]^ Xu et al. employed a precipitation method to develop mPEG‐PLGA nanoparticles encapsulating RIN, which was additionally modified with Tween‐80, for enhanced AD therapy (**Figure** **9**A).^[^ ^395^ ^]^ These nanoparticles are spherical with an average diameter of 145.2 nm, a drug loading capacity of 10.3%, and an encapsulation efficiency of 60%. The Tween 80 coating on these nanoparticles facilitated binding to apolipoprotein E, thereby enhancing RIN transport across the BBB via RMT.^[^ ^396^, ^397^ ^]^ In an in vitro BBB model, T80‐NPS‐RIN exhibited a higher cellular uptake rate and the ability to form tight connections. In vivo studies showed that, following intravenous administration, T80‐NPS‐RIN achieved the highest peak plasma concentration relative to free RIN or NPS‐RIN. Additionally, the area under the curve (AUC) for T80‐NPS‐RIN was approximately three‐fold higher than that of free RIN, with a notably lower clearance rate (Cl). Furthermore, in the apoptosis assays, all groups demonstrated a decrease in cell death, with the T80‐NPS‐RIN group exhibiting the most substantial reduction of 8.18%. These results indicate that T80‐NPS‐RIN can penetrate the brain via BBB transporters, potentially offering superior neuroprotection. Similarly, Kuo et al. formulated phosphatidylcholine liposomes infused with rosemarinic acid, CUR, epigallocatechin gallate, and QCT designed for brain‐targeted drug delivery through surface cross‐linking with glutathione and apolipoprotein E to mitigate AD by inhibiting tau protein hyperphosphorylation.^[^ ^398^ ^]^ Kuo et al. engineered a compound featuring lactoferrin‐modified RMP‐7 (a bradykinin analog) with QCT‐encapsulated liposomes, which facilitated the BBB traversal of QCT via the RMT pathway for neuronal protection in AD.^[^ ^399^ ^]^ Pinheiro et al. developed transferrin‐functionalized lipid nanoparticles (SLNs and NLCs) carrying QCT, which traversed the BBB through transferrin receptors abundantly expressed on brain endothelial cells, offering a potential AD treatment by preventing β‐amyloid aggregation.^[^ ^400^ ^]^

In addition to polymer and lipid‐based nanocarriers, biomimetic nanocarriers have gained prominence in AD therapy. Gao et al. engineered a biomimetic core‐shell nanostructure for encapsulating CUR within human serum albumin nanoparticles, which were then disguised with erythrocyte membranes. They attached T807 (a positron emission tomography imaging agent with neuronal cell‐binding specificity)^[^ ^401^, ^402^ ^]^ and triphenylphosphine (a mitochondria‐targeting ligand)^[^ ^403^ ^]^ to the membrane surface using an NHS‐amino coupling reaction for AD treatment (Figure 9B).^[^ ^404^ ^]^ The synergistic action of these two functional groups on the surface not only facilitated BBB penetration but also enhanced the targeting of neuronal mitochondria. In in vivo pharmacokinetic studies, these CUR‐loaded nanoparticles showed an extended circulation time. Neuronal targeting was evidenced by pronounced intracellular fluorescence and mitochondrial colocalization. Furthermore, to ascertain BBB penetration, live imaging revealed a substantial brain distribution of these CUR‐loaded nanoparticles, achieving a 5.6‐fold increase in brain CUR concentration compared to free CUR. Additionally, the researchers quantified oxidative stress biomarkers (SOD, H_2_O_2_, γ‐glutamyl transferase, and malionaldehyde), noting that these nanoparticles markedly decreased oxidative stress. The Morris water maze test results indicated enhanced cognitive function. These studies confirm that the biomimetic nanodelivery system is a viable therapeutic approach for AD. In a similar vein, Qi et al. crafted plasma exosomes encapsulating QCT, enhancing its bioavailability and brain targeting to alleviate cognitive deficits in AD rats through the inhibition of tau protein phosphorylation and subsequent neurofibrillary tangle formation.^[^ ^405^ ^]^ Wang et al. engineered CUR‐infused exosomes that thwarted tau protein hyperphosphorylation through the AKT/GSK‐3β pathway, thereby enhancing learning and cognitive abilities in AD mice.^[^ ^406^ ^]^

Although biomimetic nanomaterials have improved biocompatibility and safety profiles, their extraction is typically complex and costly, with low encapsulation efficiencies.^[^ ^407^ ^]^ Consequently, the proposition of a biomimetic‐based hybrid nanocarrier aims to amalgamate the merits of various nanomaterials and circumvent adverse reactions. For instance, Fernandes et al. synthesized exosome‐mimicking liposomes (exoliposomes) using a thin‐film hydration method to facilitate CUR delivery to the brain, representing a novel methodology for AD therapy (Figure 9C).^[^ ^408^ ^]^ These exosome‐analogous liposomes incorporated DODAP (1,2‐dioleoyl‐3‐dimethylammonium‐propane) to boost CUR encapsulation. Its sub‐200 nm dimensions facilitate BBB traversal via lattice protein‐mediated endocytosis.^[^ ^409^ ^]^ In neuroprotection assays using a cell model subjected to oxidative stress, the efficacy of CUR‐encapsulated exoliposomes surpassed that of free CUR. Moreover, the group treated with CUR‐encapsulated exoliposomes exhibited diminished ROS levels and decreased apoptosis rates. In summary, exoliposomes demonstrated non‐cytotoxicity and effectively mitigated apoptosis induced by oxidative stress, offering neuroprotection and a safer and more innovative therapeutic avenue for AD.

### 6.2 Parkinson's Disease

**Figure 10:**
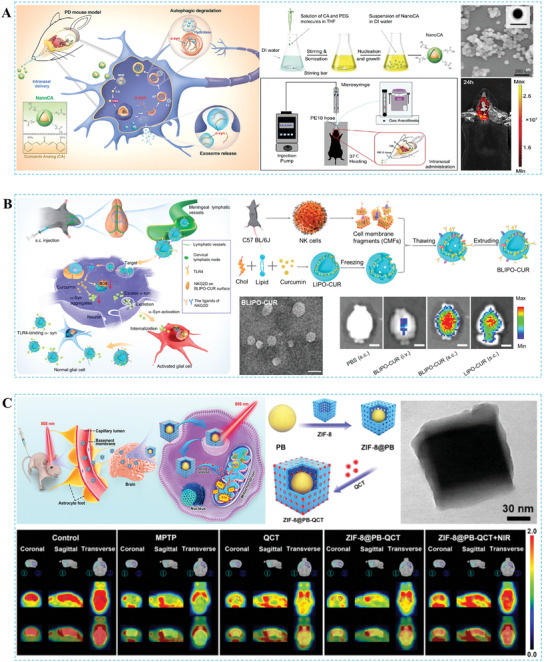
Application of nano‐TCM in PD treatment strategies. A) NanoCA was administered to the brain through the nasal route to clear α‐synuclein for PD treatment. Reproduced with permission.^[^ ^410^ ^]^ Copyright 2020, American Chemical Society. B) Brain‐targeted delivery of BLIPO‐CUR through the MLV pathway for AD treatment. Reproduced with permission.^[^ ^413^ ^]^ Copyright 2023, American Association for the Advancement of Science. C) NIR‐assisted delivery of ZIF‐8@PB‐QCT into the brain can ameliorate mitochondrial dysfunction in PD treatment. Reproduced with permission.^[^ ^415^ ^]^ Copyright 2021, American Chemical Society.

Researchers have examined multiple nanotechnology‐based methodologies for the treatment of PD to enhance drug delivery precision and therapeutic efficacy. Liu et al. employed a reprecipitation technique to synthesize a self‐assembling nanoformulation (NanoCA) composed of CUR analogs (CA) and PEG intended for brain‐targeted PD therapy via nasal administration (**Figure** **10**A).^[^ ^410^ ^]^ This CA can selectively bind to the basic helix‐loop‐helix transcription factor EB (an autophagy regulator)^[^ ^411^ ^]^ and activate the target, thereby promoting autophagic degradation and exosomal release of α‐syn for neuroprotection. NanoCA possesses a particle size of 131.1 nm, a drug load capacity of 26.95%, and exhibits outstanding slow‐release and stabilization characteristics. In vitro, NanoCA enhanced cellular autophagy and facilitated exosome‐mediated clearance of α‐syn. In vivo, after intranasal administration in mice, CA levels in the olfactory bulb tissue and CSF increased, indicating the drug entered the olfactory bulb directly through the olfactory nerve and diffused to distant parts of the brain via the CSF. Moreover, the nasal administration of NanoCA ameliorated memory impairment, gait disorders, and anxiety. Furthermore, in the NanoCA group, there was a higher density of olfactory bulb neurons with elevated expression of tyrosine hydroxylase (TH), a biomarker of dopaminergic neurons, and a significant increase in striatal dopaminergic fibers and TH‐positive neurons. In summary, nasally administered NanoCA demonstrated significant neuroprotection by clearing α‐syn from the brain. This noninvasive approach offers a promising option for long‐term PD treatment. Similarly, Chen et al. fabricated a slow‐release mPEG‐PLGA polymer nanomedicine containing Schisantherin A to enhance the BBB penetration, brain uptake, and anti‐Parkinsonian activity of the drug.^[^ ^412^ ^]^

Compared to nanocarriers such as the aforementioned polymers, biomimetic nanocarriers hold greater promise in PD therapy owing to their superior biocompatibility and safety profile. Liu et al. formulated CUR‐loaded biomimetic liposomes (BLIPO‐CUR) through a process of repeated freeze‐thaw cycles involving CUR‐loaded liposomes and natural killer (NK) cell membrane fragments from mice and subsequently administered them via meningeal lymphatic vessels (MLVs) for targeted PD therapy (Figure 10B).^[^ ^413^ ^]^ NK cell‐modified BLIPO‐CUR is capable of transitioning from the cervical lymph nodes to MLVs, thereby circumventing phagocytosis to achieve brain‐targeted neuroprotection. The particle size of BLIPO‐CUR was determined to be approximately 82 nm (permeable to lymphatic vessels), with a drug loading efficiency (LE) and encapsulation efficiency (EE) measured at 12.6 ± 0.3% and 66.7 ± 2.1%, respectively. The delivery efficiency of biomimetic liposomes through the MLV pathway to the brain is approximately 20 times that of the brain–blood system. In vitro experiments demonstrated that the rates of apoptosis and the number of ROS‐positive cells were significantly diminished by BLIPO‐CUR. In vivo, the formulations were administered subcutaneously to mice via the cervical lymph nodes. Fluorescence imaging confirmed that BLIPO‐CUR successfully reached the brain via the MLV pathway. Behavioral assessments validated the efficacy of BLIPO‐CUR, which improved memory and alleviated anxiety in PD mice. Histological analysis showed enhanced survival of dopaminergic neurons, along with reduced ROS and α‐syn levels in the group that received subcutaneous injections of BLIPO‐CUR compared with the control group. The aforementioned experiments illustrate that BLIPO‐CUR possesses enhanced immune evasion and brain‐targeting capabilities and can effectively deliver the active constituents of TCM through the MLV pathway for the treatment of PD. Similarly, Liu et al. prepared biomimetic nanoparticles for the targeted therapy of PD utilizing membranes from substantia nigra dopaminergic neurons (MES23.5 cells) modified with QCT‐containing poly(vinylpyrrolidone).^[^ ^414^ ^]^ The emergence of such biomimetic nanocarriers could provide a novel pathway and concept for PD treatment.

In recent years, with the expanding applications of nanotechnology in PD therapy, there has been growing interest in employing extracorporeal physical devices as adjunct modalities to enhance BBB permeability. Yao et al. synthesized a zeolitic imidazolate framework‐8 coated with a Prussian blue nanocomposite containing QCT (ZIF‐8@PB‐QCT) using a vacuum freeze‐drying method, which was directed through the BBB using NIR and applied it in PD treatment to ameliorate mitochondrial dysfunction and reduce ROS levels (Figure 10C).^[^ ^415^ ^]^ The ZIF‐8@PB nanoparticles were cubes with an average size of 107 nm, whereas the ZIF‐8@PB‐QCT nanoparticles displayed fuzzy boundaries and were larger in size. This porous structure facilitated a high drug loading capacity of 23.5% and an encapsulation efficiency of 61.3%. Under an 808 nm NIR laser, the temperature of the solution increased from 33.0 °C to 48.2 °C, which enhanced the release of QCT from 13% to 77.96%, underscoring the excellent photothermal properties of NIR‐induced drug release. In vitro, the permeability of ZIF‐8@PB‐QCT increased by 1.67‐fold, which further increased by 2.67‐fold upon NIR exposure. Additionally, ZIF‐8@PB‐QCT+NIR irradiation significantly reduced apoptosis and ROS levels. In vivo, the ZIF‐8@PB‐QCT+NIR group exhibited the greatest brain accumulation in vivo, as evidenced by a 4.3‐fold increase in the AUC_0‐t_ value. Furthermore, treated PD mice demonstrated significant improvements in motor function and brain glucose metabolism, increased TH+ neuron levels, and potential normalization of the indicators of inflammation and mitochondrial function. In summary, the integration of nanotechnology and NIR significantly improves the penetration of TCM. This noninvasive, targeted therapy involving physical devices and nano‐TCM holds promise for future PD treatment.

### 6.3 Stroke

**Figure 11:**
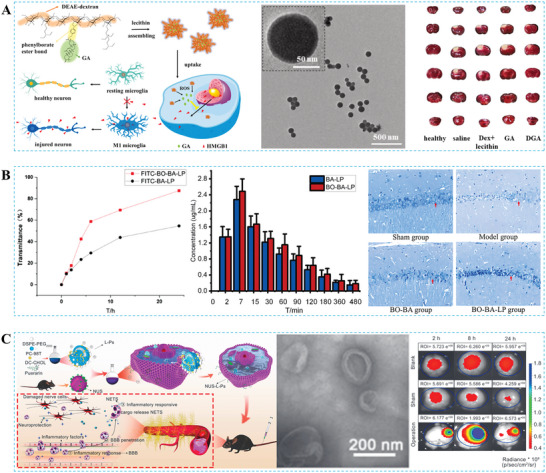
Application of nano‐TCM treatment strategies in stroke. A) DGA nanoparticles with ROS‐responsive function regulate microglia polarization by inhibiting HMGB1 secretion and play a neuroprotective role in ischemic stroke. Reproduced with permission.^[^ ^417^ ^]^ Copyright 2023, Elsevier. B) Brain‐targeted BO‐BA‐LP can treat CIRI by inhibiting the HIF‐1α/VEGF/eNOS/NO signaling pathway. Reproduced with permission.^[^ ^421^ ^]^ Copyright 2023, Elsevier. C) NUS‐L‐Ps cross the BBB to treat CIRI with the help of the electrostatic interaction of cationic liposomes and the inflammatory chemotaxis of neutrophils. Reproduced with permission.^[^ ^425^ ^]^ Copyright 2021, Tsinghua Press, co‐published with Springer‐Verlag GmbH.

During cerebral ischemia, high‐mobility group box 1 (HMGB1) binds to microglia, promoting M1 polarization and exacerbating inflammation.^[^ ^416^ ^]^ However, 18 β‐glycyrrhetinic acid (GA) derived from *Glycyrrhiza glabra* inhibits HMGB1 intranuclear translocation, thereby mitigating proinflammatory responses.^1^ Jin et al. developed ROS‐responsive PNs of GA conjugated to diethylaminoethyl‐dextran (DGA) using a self‐assembly method, aiming to modulate microglial polarization and exert neuroprotective effects in ischemic stroke by inhibiting HMGB1 secretion (**Figure** **11**A).^[^ ^417^ ^]^ DGA nanoparticles are homogeneous and spherical, with a particle size of 104.8 nm. In vitro, a reduction in HMGB1 and increased CD16/32 expression were observed in the cell model group, whereas DGA preserved HMGB1 within the nucleus and decreased CD16/32 fluorescence intensity by 56.0%. These findings were corroborated by in vivo staining of injured brain cells. In the stroke model mice, the DGA group displayed the smallest brain infarct area (7.0%), enhanced spontaneous neuronal maturation and regeneration in the damaged regions, and significant improvements in behavioral tests. In conclusion, a nanodelivery strategy using DGA is an effective treatment approach for stroke. Similarly, Yang et al. developed CUR‐loaded PNs to treat cerebral hemorrhage by inhibiting ferroptosis.^[^ ^418^ ^]^ Lu et al. encapsulated resveratrol in PNs and combined it with endovascular thrombectomy to treat CIRI in aortic occlusion strokes.^[^ ^419^ ^]^ Shen et al. developed ginsenoside Rg1‐loaded PNs to treat cerebral infarction complicated by diabetes mellitus using receptor‐mediated transferrin transport across the BBB.^[^ ^420^ ^]^

Notably, these nanocarriers can be combined with specific herbal medicines that enhance BBB permeability, thereby offering additional therapeutic options for stroke treatment. Long et al. prepared borneol‐baicalin liposomes (BO‐BA‐LPs) using a reverse evaporation method to treat CIRI (Figure 11B).^[^ ^421^ ^]^ Baicalin, an effective flavonoid for treating CIRI, has clinical limitations owing to its low solubility and difficulty crossing the BBB. However, borneol enhances BBB permeability and improves brain targeting. In vitro, BO‐BA‐LPs demonstrated significantly higher transmittance (87.49%) compared to BA‐LP (54.65%), confirming borneol's efficacy in enhancing baicalin's BBB penetration. In vivo, BO‐BA‐LPs displayed higher AUC values and maximum baicalin concentrations in both the plasma and brain tissues than the other groups. Moreover, the BO‐BA‐LP group demonstrated the most favorable outcomes in behavioral scores and cerebral edema measurements, with a significant reduction in neuronal necrosis and an increase in Nissl bodies within the injured area upon pathological examination, indicating a superior protective effect on brain tissue. Similarly, Zhang et al. utilized BO‐BA‐LPs to enhance BBB permeability and pharmacokinetics and ameliorate CIRI.^[^ ^422^ ^]^ Lv et al. modified ginkgolide liposomes with borneol to increase BBB permeability and brain uptake, targeting the treatment of ischemic stroke and cerebral infarction.^[^ ^423^ ^]^ Yuan et al. developed a nanostructured lipid carrier incorporating tanshinol borneol ester with PEG modification, which was intended to improve sustained release, BBB permeability, and antioxidant activity for the treatment of CIRI.^[^ ^424^ ^]^ In conclusion, leveraging the brain‐targeting properties of borneol, along with the physicochemical characteristics of nanocarriers, presents a promising approach for enhancing drug delivery through the BBB, which is potentially applicable to stroke and other CNS disorders.

Given the pathological mechanism of stroke, a tailored nanodelivery strategy can more effectively target stroke treatment. Liu et al. engineered a “Trojan horse” system, loading puerarin into cationic liposomes with neutrophils serving as carriers (NUS‐L‐Ps), based on the CIRI‐induced inflammatory response (Figure 11C).^[^ ^425^ ^]^ Puerarin, an isoflavone derivative from *Pueraria lobata*, has antioxidant and ROS‐scavenging effects but has low solubility and BBB permeability.^[^ ^426^ ^]^ Employing the electrostatic effects of L‐Ps for higher drug loading and inflammatory factor chemotaxis of neutrophils to transport puerarin across the BBB optimized their neuroprotective potential. L‐Ps are uniformly spherical with a particle size of 107.26 ± 0.55 nm, a zeta potential of 33.30 ± 1.93 mV, and a drug loading capacity of 2.22 ± 0.57%. In vitro, NUS‐L‐Ps showed limited penetration under non‐inflammatory conditions but significantly higher trans‐BBB concentrations under inflammation. In vivo, the fluorescence intensity in the brains of the NUS‐L‐P group was three times higher than that of the control group after 8 h and primarily concentrated in the ischemic hemisphere. Furthermore, NUS‐L‐Ps markedly reduced the infarct area, and behavioral assessments showed significant improvements in spatial learning, motor function, and memory. In conclusion, NUS‐L‐Ps can effectively respond to inflammatory chemokines, actively target the site of brain injury, and effectively treat CIRI. This design concept, which involves selecting various nanocarriers for combined use based on the application challenges of TCM and the pathological features of CIRI, offers a promising strategy for drug delivery in the brain‐targeted treatment of ischemic stroke.

### 6.4 Brain Tumors

**Figure 12:**
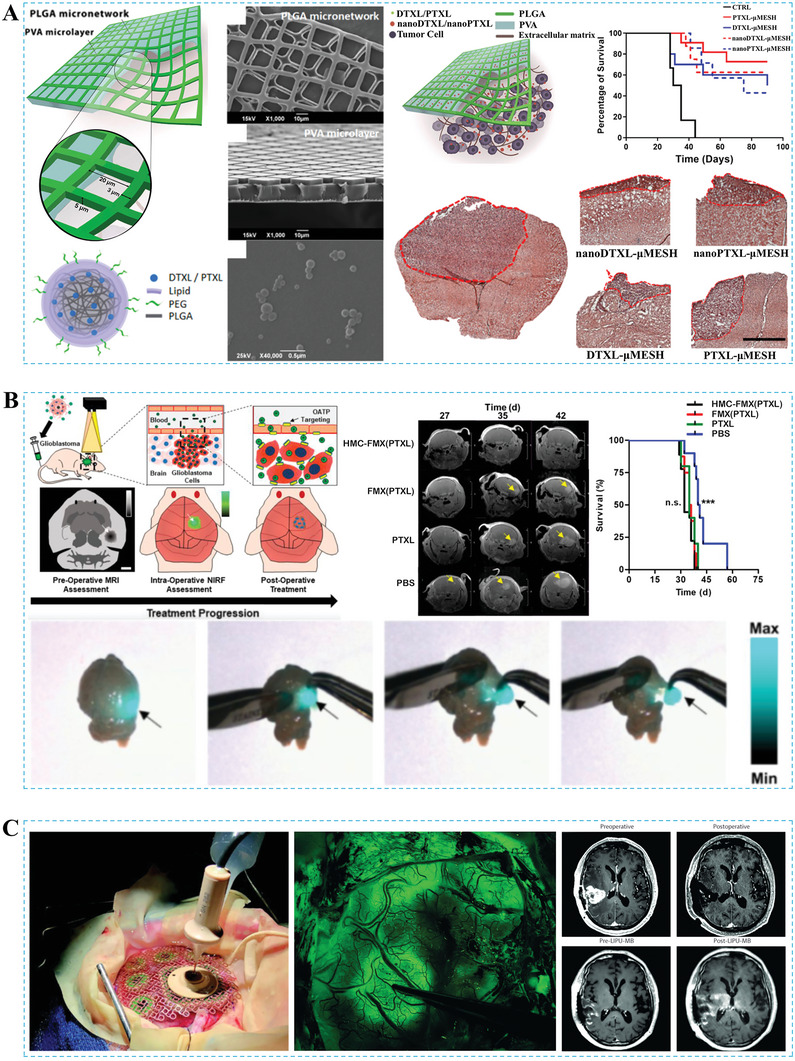
Application of nano‐TCM treatment strategies in glioma. A) An integrable polymeric nanocarrier µMESH for the sustained delivery of anti‐GBM therapeutics PTXL and DTXL. Reproduced with permission.^[^ ^427^ ^]^ Copyright 2023, American Chemical Society. B) HMC‐FMX (PTXL) combined with NIRF on a fluorescent nanoparticle platform for targeted drug delivery and visualization during intraoperative resection of GBM. Reproduced with permission.^[^ ^431^ ^]^ Copyright 2020, American Chemical Society. C) Delivery of ABX for recurrent GBM through repeated disruption of the BBB using an implantable ultrasound device (LIPU‐MB). Reproduced with permission.^[^ ^433^ ^]^ Copyright 2023, Elsevier.

Researchers have rigorously pursued advancements to improve the efficacy of polymer nanocarriers for brain cancer therapy. Di Mascolo et al. engineered an integrable polymeric nanocarrier using a sonication/evaporation method, µMESH, for the prolonged delivery of anti‐GBM therapeutic agents PTXL and DTXL (**Figure** **12**A).^[^ ^427^ ^]^ This µMESH was constructed by embedding a PLGA micronetwork on a polyvinyl alcohol microlayer. Four distinct therapeutic formulations were developed, each demonstrating sustained drug release for a minimum of 150 days. In vivo, the survival rates of the GBM model mice treated with these formulations were significantly enhanced, with the median survival in the nanoPTXL‐µMESH group increasing from 32.5 to 75 days, and reaching 90 days in the PTXL‐µMESH group. Furthermore, 60% and 80% of the mice in the nanoDTXL‐µMESH and DTXL‐µMESH groups, respectively, survived the 90‐day observation period. Additionally, analysis of brain tissue sections revealed a reduction in malignant regions in all µMESH‐treated groups when compared to the control group. In conclusion, the sustained delivery of anticancer drugs using integratable PNs µMESH can effectively prevent GBM progression. Similarly, Chen et al. modified PTX‐loaded PEG‐PLA PNs with CPPs to enhance the brain‐targeting capability of PTXL for the treatment of gliomas.^[^ ^428^ ^]^ Sun et al. synthesized PTXL‐loaded transferrin receptor‐targeting PEG‐PLA polymer micelles for glioma therapy.^[^ ^429^ ^]^ Liu et al. synthesized a QCT‐infused polyphenolic polymer designed to inhibit brain tumor growth by disrupting the tumor vasculature and diminishing neovascularization.^[^ ^430^ ^]^

Recent advancements in drug delivery platforms combining nanotechnology with physical devices have significantly advanced targeted brain cancer treatment research. Reichel et al. used a dialysis method to develop a visualized fluorescent nanoparticle platform for guided drug delivery in GBM utilizing near‐infrared fluorescence (NIRF). It consisted of ferumoxytol (FMX) loaded with the antitumor drug PTXL conjugated to heptamethine carbocyanine (HMC) (Figure 12B).^[^ ^431^ ^]^ FMX, an MRI‐sensitive superparamagnetic iron oxide nanoparticle, in conjunction with HMC, a NIRF ligand that targets organic anion‐transporting polypeptides overexpressed in GBM, merges magnetic and fluorescent properties to facilitate effective delivery of PTXL across the BBB into tumor tissues. In the *ex vivo* brains of mice injected with HMC‐FMX, intense fluorescence was observed in the region of GBM tumors, whereas fluorescence in other areas was minimal. This indicates that HMC‐FMX can penetrate the BBB and target GBM tumors in situ in mice. In vivo, the HMC‐FMX (PTXL) group exhibited higher IC50 values compared to the other groups along with a marked reduction in cell viability (56.4%) and an increase in both early (28.0%) and late (12.1%) apoptotic cells. Furthermore, HMC‐FMX (PTXL) significantly reduced the viability and spheroid‐forming ability of migratory GBM cancer stem cells (CSCs) originating from GBM patient‐derived cell lines. In vitro, HMC‐FMX (PTXL) significantly inhibited tumor growth and extended median survival by 28%. In conclusion, HMC‐FMX (PTXL) can cross the BBB to penetrate GBM tissues and accumulate persistently, thereby effectively reducing tumor volume and enhancing survival rates. Additionally, HMC‐FMX can serve as a fluorescent probe to label tumors and facilitate visual resection owing to its fluorescent imaging capabilities. Similarly, Will et al. developed a novel tLyP‐1‐modified, dopamine‐β‐cyclodextrin‐coated nanoparticle system loaded with PTXL and manganese dioxide for glioma therapy.^[^ ^432^ ^]^ On one hand, the nanoparticles enabled real‐time tumor monitoring through MRI. In contrast, the nanoparticles mitigated hypoxia‐mediated chemoresistance through the release of O_2_ by MnO_2_ and facilitated the delivery of PTXL across the BBB directly to the tumor site, thereby potentiating the chemotherapeutic effect.

Furthermore, the integration of bionanotechnology with physical devices offers an enhanced potential. Sonabend et al. utilized low‐intensity pulsed ultrasound with the concurrent administration of intravenous microbubbles (LIPU‐MB) to open the BBB in combination with an albumin‐bound PTXL formulation (ABX) for the treatment of recurrent GBM (Figure 12C).^[^ ^433^ ^]^ Intravenously injected microbubbles, when stimulated by ultrasound, generate mechanical stress on the endothelial walls of the brain capillaries, thereby opening the BBB to enhance permeability. Previous studies have observed that ultrasound combined with ABX can increase PTXL concentration and prolong survival.^[^ ^434^ ^]^ A phase 1 clinical trial involved implanting a nine‐emitter ultrasound device in patients with recurrent GBM after resection and administering variable intravenous doses of ABX. Imaging analyses one‐hour post‐ultrasound revealed BBB disruption in LIPU‐MB‐targeted brain regions. Pharmacokinetic analysis indicated that the mean PTXL concentration in the brain was 3.7 times higher than that in the non‐ultrasound group. In conclusion, LIPU‐MBs can effectively disrupt the BBB, facilitating the safe and repeated delivery of anticancer drugs into the brain for recurrent GBM treatment. Similarly, Wang et al. developed a PTX‐containing biomimetic hypoxia‐triggered RNA interference nanomedicine to synergistically enhance chemotherapy and radiotherapy for the treatment of glioma.^[^ ^435^ ^]^ Meng et al. engineered an extracellular vesicular bionic nanoparticle encapsulating PTX and indirubin green combined with NIRF imaging for guided hyperthermia and chemotherapy for the treatment of glioma.^[^ ^436^ ^]^

### 6.5 Other CNS Diseases

**Figure 13:**
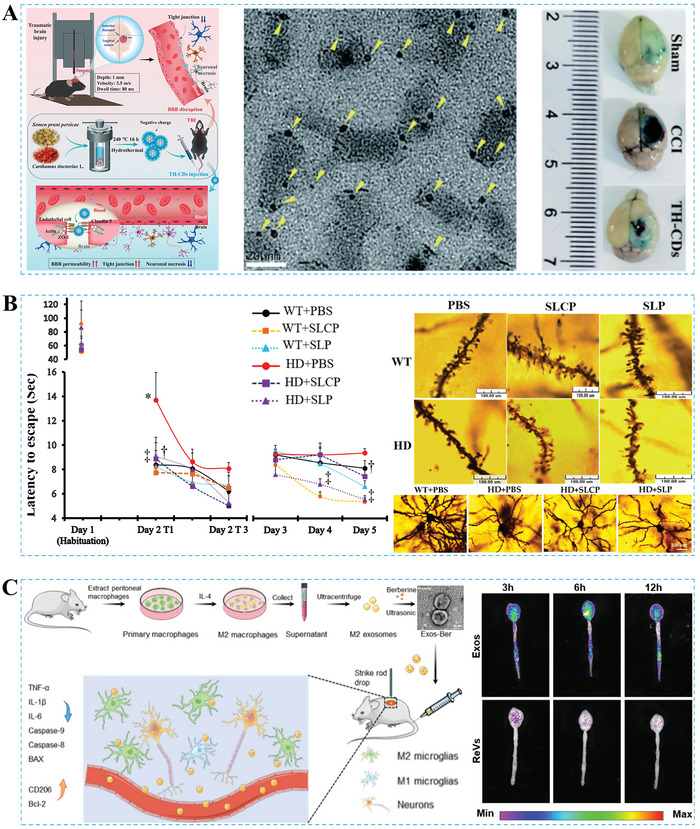
Application of nano‐TCM treatment strategies in other CNS diseases. A) Intravenous injection of green, non‐toxic, functionally negatively charged TH‐CDs to repair the BBB for TBI treatment. Reproduced with permission.^[^ ^437^ ^]^ Copyright 2022, Tsinghua Press, co‐published with Springer‐Verlag GmbH. B) SLCPs protect the morphology of medium spiny neurons and ameliorate memory and learning deficits in HD model mice. Reproduced with permission.^[^ ^438^ ^]^ Copyright 2020, MDPI. C) Exos‐Ber treats SCI by promoting the polarization of the M1 phenotype to the M2 phenotype. Reproduced with permission.^[^ ^334^ ^]^ Copyright 2021, Elsevier.

In addition to the four prevalent CNS diseases mentioned above, nano‐TCM technology has been applied to the treatment of various other CNS disorders. Luo et al. used a dialysis method to synthesize novel green and non‐toxic negatively charged Taoren‐Honghua carbon dots (TH‐CDs) for intravenous use, employing hydrothermal synthesis to treat traumatic brain injury (TBI) (**Figure** **13**A).^[^ ^437^ ^]^ TBI often results in disruption of the BBB, allowing undesired molecules to enter the brain tissue and cause pathological changes, including neuroinflammation, neuronal necrosis, and cerebral edema. Most patients with TBI experience a loss of consciousness and require rapid intervention. Taoren and Honghua exhibit neuroprotective effects against TBI; however, their limited oral bioavailability restricts their clinical use. TH‐CDs address the first‐pass effect through intravenous injection and repair the BBB for TBI treatment via AMT, facilitated by electrostatic interaction with claudin5, with particle sizes ranging from 2–5 nm and a zeta potential of −29.7 mV. In vivo, mice with TBI injected with TH‐CDs exhibited reduced neurological impairment, decreased cerebral water content, and mitigated neuronal damage, including nuclear atrophy, reduced cell volume, and disruption of Nissl bodies. Additionally, both Evans blue staining and pathological extravasation of IgG were significantly reduced in the TH‐CDs group, and the expression of ZO‐1 and claudin‐5 was upregulated. In conclusion, TH‐CDs are not only green and non‐toxic, but also effective in improving neuronal damage, reducing brain edema, enhancing neurological function, and repairing damaged BBB in the pathological process of TBI.

Gharaibeh et al. developed CUR‐containing solid lipid nanoparticles (SLCPs) to ameliorate neurological deficits in Huntington's disease (HD), a hereditary neurodegenerative disorder characterized by cognitive, psychiatric, and motor symptoms (Figure 13B).^[^ ^438^ ^]^ The primary pathological mechanism involves the deletion of brain‐derived neurotrophic factor (BDNF) and alterations in its receptor (TrkB) in medium spiny neurons (MSNs). Additionally, the substantial release of the postsynaptic membrane protein PSD‐95 disrupts synaptic transmission and leads to corresponding alterations in neurological function. In an active avoidance task, mice administered SLCPs via gavage for eight weeks demonstrated significantly reduced avoidance latency. Golgi‐Cox staining showed that the number and length of dendritic branches, as well as the density of dendritic spines in MSNs, were significantly greater in the SLCP‐treated group than in the other groups. Furthermore, in western blot analyses, BDNF and TrkB levels were increased, while PSD95 levels were decreased in the SLCP‐treated group. In conclusion, this study suggests that SLCPs can alleviate the pathological and cognitive deficits in HD mice, offering significant potential for future HD treatments.

Gao et al. employed an ultrasound technique to produce berberine‐loaded M2 macrophage‐derived exosomes (Exos‐Ber) for the treatment of spinal cord injury (SCI) (Figure 13C).^[^ ^334^ ^]^ In SCI, an irreversible loss of motor neurons occurs, leading to sensory and motor dysfunction, which severely affects the patient's quality of life. A key aspect of the SCI secondary injury mechanism is the inflammatory response, which involves the immune activation of macrophages/microglia and the activation of a predominantly proinflammatory, classically activated (M1) phenotype. Inflammation can be effectively mitigated by promoting the polarization of the M1 phenotype to an anti‐inflammatory alternatively activated (M2) phenotype. Exosomes serve as ideal nanocarriers for SCI therapy by delivering berberine, which possesses anti‐inflammatory, antioxidant, and neuroprotective properties while overcoming the limitations of the BBB and immune response. Exos‐Ber particles are 125 ± 12 nm in size, with a drug loading rate of 17.13 ± 1.64% and a cumulative release of 71.44% at 48 hours. In the in vitro BBB model, the cellular uptake of Exos‐Ber was 18.27 times higher than that of the control group. Exos‐Ber clearly targeted injured brain and spinal cord tissues. Additionally, Exos‐Ber downregulated the M1 protein marker iNOS and upregulated the M2 protein marker CD206, indicating a shift toward M2 polarization in macrophage/microglial cells. Furthermore, Exos‐Ber reduced the levels of inflammatory factors, including TNF‐α, IL‐1β, and IL‐6, as well as apoptotic cytokines such as Caspase 9 and Caspase 8. Moreover, SCI mice treated with Exos‐Ber exhibited the highest rates of neuronal survival and the most significant recovery of motor function. In conclusion, Exos‐Ber, a novel bionic nanodelivery system, is a promising therapeutic option for SCI treatment.

## 7 Conclusions and Future Perspectives

One of the greatest challenges faced by TCM researchers and practitioners is overcoming BBB permeability to achieve effective treatment of CNS diseases. However, with the advent of nano‐TCM, new and more efficient brain‐targeted drug delivery systems have been developed. Owing to the physiological structure and mechanisms of the BBB, it is uncommon for nanosized or modified TCM compounds to pass directly through the BBB. Most TCM agents depend on the specific properties, physical structures, and functional modifications of the nanocarriers to ensure effective traversal of the BBB (Table 2). Nevertheless, the safety of the nano‐TCM preparation process requires further improvement, and key issues such as the targets of action and related pharmacological mechanisms warrant further investigation. Future research will focus on the following aspects to develop optimal nano‐TCM delivery strategies for the treatment of CNS diseases.

From a biosafety perspective, the stability and safety of artificially prepared nanocarriers present concerns.^[^ ^439^ ^]^ For instance, drug‐loaded nanocarriers maintain structural stability while circulating in the body before reaching their target sites. Alternatively, there may be a toxic or non‐degradable accumulation of nanocarriers within the body. In this scenario, the optimal solution appears to be the development of biomimetic nanomaterials with enhanced biocompatibility and safety, utilizing endogenous substances in the human body as carriers, such as the aforementioned proteins and cells. Moreover, the targeting efficacy, biosafety, and therapeutic attributes of nanomedicine can be enhanced by advancing the engineering of precision nanoparticles characterized by meticulously tailored surfaces and structural heterogeneity.^[^ ^440^ ^]^

From the perspective of pathomechanisms, new biomimetic nanomaterials can be developed for targeted drug delivery to the brain through in‐depth research on CNS diseases. For instance, conditions such as rabies and melanoma, which are prone to brain metastasis at later stages, have been found to achieve BBB permeability with associated viral proteins or secreted exosomes, which enter the brain and invade the CNS.^[^ ^441^, ^442^ ^]^ Similarly, “Trojan horse” approaches could be employed by encapsulating TCM within biomimetic nanomaterials. Additionally, modifying a single functional group or using only one type of nanocarrier often fails to yield satisfactory results. A design concept that involves selecting multiple functional groups for modification and combining various forms of nanocarriers based on the pathological mechanisms of the disease represents the most promising nano‐TCM drug delivery system for the future.

Regarding performance, TCM use is not straightforward, owing to the different needs and characteristics of diseases. Aromatic TCMs such as borneol, musk, and mint, which enhance BBB permeability, may be useful for CNS diseases such as glioma, AD, and PD. Conversely, there are conditions in which the BBB is compromised, necessitating prompt repair. For instance, in heat stroke, the BBB can become damaged, leading to brain edema; thus, an appropriate TCM is needed to diminish its permeability, ensure maintenance and stability, and repair the damage as extensively as possible to optimally protect brain tissue. Numerous TCMs ameliorate BBB dysfunction, including ruscogenin, schisandrin A, and ginsenoside Rb1, which are found in Shengmai powder.^[^ ^443^ ^]^ Notably, the aforementioned TCMs have not been extensively developed to incorporate nanotechnology. In the future, the potential of TCMs for treating CNS diseases could be explored more comprehensively by enhancing the drug load and bioavailability of these TCMs using suitable nanocarriers.

In summary, with the aid of nanotechnology, TCM presents improved prospects for the development and application of treatments for CNS damage‐related diseases. We believe that in the future, with the rapid advancement of nanotechnology and emerging research breakthroughs, the vast potential of nano‐TCM in BBB applications can be harnessed to unveil more promising therapeutic avenues for CNS damage‐related diseases.

## Conflict of Interest

The authors declare no conflict of interest.
